# CO_2_ electroreduction to multicarbon products in strongly acidic electrolyte via synergistically modulating the local microenvironment

**DOI:** 10.1038/s41467-022-35415-x

**Published:** 2022-12-09

**Authors:** Zesong Ma, Zhilong Yang, Wenchuan Lai, Qiyou Wang, Yan Qiao, Haolan Tao, Cheng Lian, Min Liu, Chao Ma, Anlian Pan, Hongwen Huang

**Affiliations:** 1 College of Materials Science and Engineering, Hunan University, Changsha, Hunan 410082 China; 2 State Key Laboratory of Powder Metallurgy, School of Physical and Electronics, Central South University, Changsha, Hunan 410083 China; 3 State Key Laboratory of Chemical Engineering and Shanghai Engineering Research Center of Hierarchical Nanomaterials, School of Chemistry and Molecular Engineering, East China University of Science and Technology, Shanghai, 200237 China; 4 Shenzhen Research Institute of Hunan University, Shenzhen, Guangdong 518055 China

**Keywords:** Electrocatalysis, Electrocatalysis, Energy

## Abstract

Electrochemical CO_2_ reduction to multicarbon products faces challenges of unsatisfactory selectivity, productivity, and long-term stability. Herein, we demonstrate CO_2_ electroreduction in strongly acidic electrolyte (pH ≤ 1) on electrochemically reduced porous Cu nanosheets by combining the confinement effect and cation effect to synergistically modulate the local microenvironment. A Faradaic efficiency of 83.7 ± 1.4% and partial current density of 0.56 ± 0.02 A cm^−2^, single-pass carbon efficiency of 54.4%, and stable electrolysis of 30 h in a flow cell are demonstrated for multicarbon products in a strongly acidic aqueous electrolyte consisting of sulfuric acid and KCl with pH ≤ 1. Mechanistically, the accumulated species (e.g., K^+^ and OH^−^) on the Helmholtz plane account for the selectivity and activity toward multicarbon products by kinetically reducing the proton coverage and thermodynamically favoring the CO_2_ conversion. We find that the K^+^ cations facilitate C-C coupling through local interaction between K^+^ and the key intermediate *OCCO.

## Abstract (web summary)

Attaining high selectivity for CO_2_ electroreduction in acid is usually difficult due to competing hydrogen evolution. Here, the authors demonstrate efficient CO_2_ reduction to multicarbon products in strongly acidic medium (pH ≤ 1) on a porous Cu catalyst by combining confinement and cation effects.

## Introduction

Electrochemical CO_2_ reduction reaction (CO_2_RR) powered by clean electricity provides a promising route to lower CO_2_ atmospheric concentration and simultaneously store the intermittent renewable energy in chemicals^1,2^. Among diverse products from CO_2_RR, multicarbon (C_2+_) products are of particular interest due to their high-energy-density feature and important role in modern chemical industry^3,4^. Towards the C_2+_ production, copper (Cu) is the unique catalyst with moderate adsorption of *CO intermediate, but faces the dilemma of the poor selectivity and productivity due to the competing hydrogen evolution reaction (HER) and the involved complex reaction pathways for CO_2_RR^5–7^.

Presently, thermodynamically optimizing the adsorption of *CO intermediate on active sites, kinetically facilitating the C–C coupling, and suppressing the HER process represent the basic principles to design the efficient catalytic system for C_2+_ formation^8–10^. Under the guidance of these principles, alkaline electrolyte has become the mainstream choice because competing HER usually dominates in acidic solutions, and high-alkalinity microenvironment can further facilitate the C–C coupling step^11,12^. So far, great advances have been made to enhance the C_2+_ selectivity and productivity via constructing the favorable structure of Cu-based catalysts and modulating the microenvironment in the vicinity of catalyst surface^13–15^. However, beyond the unsatisfactory selectivity and productivity, low carbon utilization efficiency and long-term stability issue associated with the carbonate formation in alkaline system have greatly impeded the practical viability of alkaline CO_2_ electrolysis^16–18^.

Very recently, acidic CO_2_ electrolysis has been developed to resolve the carbonate problem in alkaline system, where acidic electrolyte is utilized to avoid the formation of carbonate, or to recover the CO_2_ reactant from locally generated carbonate^19–25^. The pioneering work by Sargent and co-workers has demonstrated that a well-designed acidic CO_2_RR system could achieve C_2+_ products with high carbon utilization efficiency and good electrolysis stability because of the carbonate inhibition^21^. However, the kinetically more favorable HER process greatly limits the C_2+_ selectivity under strongly acidic media especially when pH ≤ 1, with the optimal C_2+_ selectivity of 48% reported at present^21^, which is still far from the industrial requirements. Therefore, it is still a big challenge to further suppress HER and realize efficient C_2+_ production in acidic CO_2_RR system^24,26,27^.

Herein, we attempt to design an efficient catalytic system for C_2+_ formation by synergistically modulating the reaction microenvironment at the catalyst-electrolyte interface. It is recognized the C_2+_ selectivity and activity strongly depend on the local microenvironment, which determines the reactive/non-reactive species (e.g., proton, OH^−^, *CO) distribution on the Helmholtz plane and local interactions between those species^24,28,29^. Specifically, we actualize a highly selective, efficient, and stable CO_2_RR in strongly acidic electrolyte (pH ≤ 1) over the porous Cu nanosheets via combining the confinement effect and cation effect. Consequently, a high Faradaic efficiency (FE) of 83.7 ± 1.4%, large partial current density of 0.56 ± 0.02 A cm^−2^, and high single-pass carbon efficiency (SPCE) of 54.4% and stable electrolysis of 30 h, are actualized for C_2+_ products. The mechanistic studies performed by a combination of theoretical simulations and experiments well rationalize the improved selectivity and activity toward C_2+_ products, where the cation effect on promoting C–C coupling kinetics is a crucial factor.

## Results

### Synthesis and characterization

**Fig. 1:**
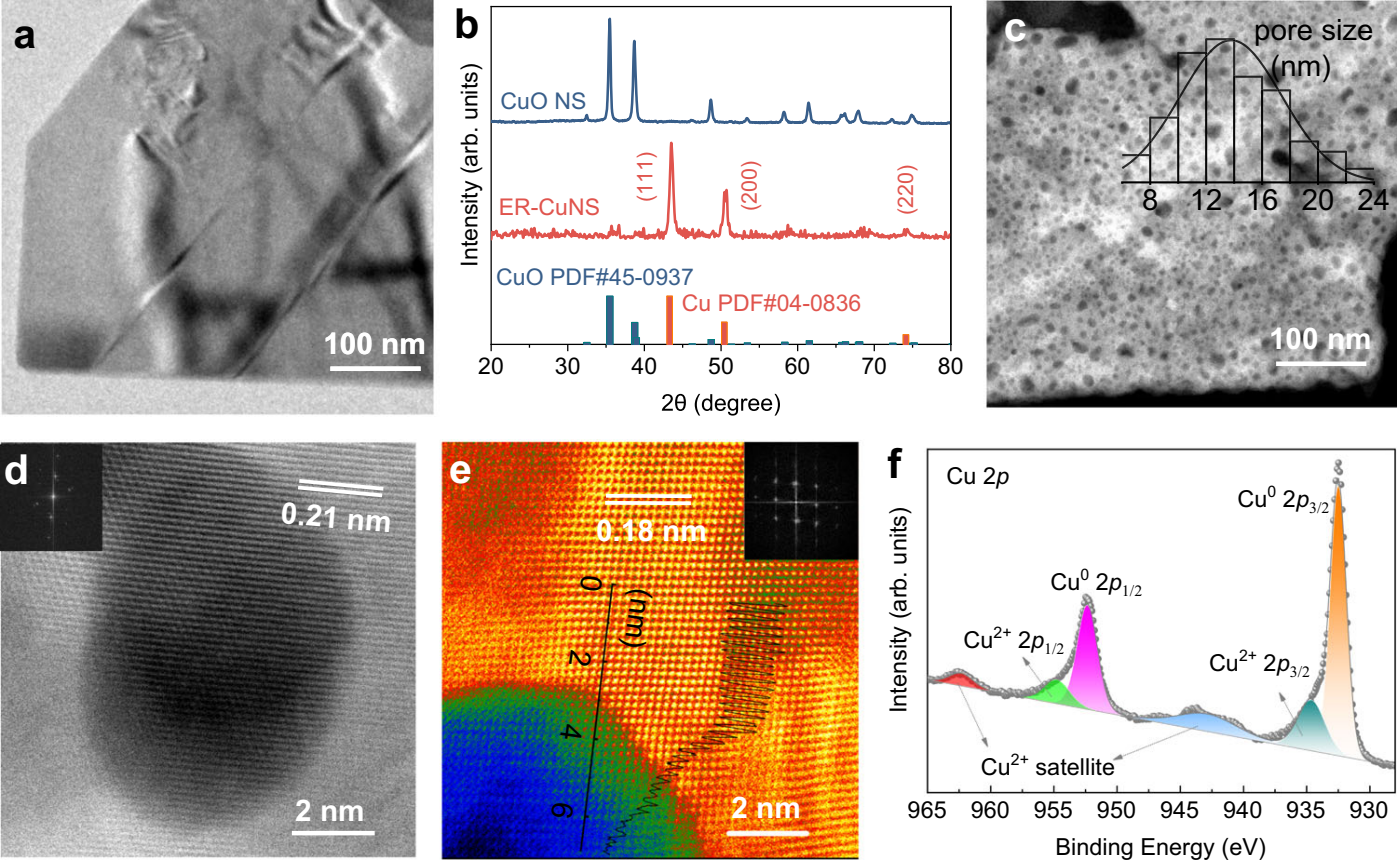
Structural characterizations. **a** Low-magnification TEM image of as-prepared CuO NS. **b** XRD patterns of CuO NS and ER-CuNS with standard PDF cards of CuO and Cu. **c** HAADF-STEM image of ER-CuNS. The inset is the size distribution of pores. **d**, **e** Atomic-resolution aberration-corrected HAADF-STEM image of ER-CuNS with FFT shown in insets. The trace in (**e**) reflects the height profile. **f** XPS Cu 2*p* spectrum of ER-CuNS.

The electrochemically reduced Cu porous nanosheet (ER-CuNS) was fabricated through a two-step process (see “Methods” for details), as schematically illustrated in Supplementary Fig. 1. The CuO nanosheet (CuO NS) was first obtained via a hydrothermal reaction of Cu precursor in a Teflon-lined autoclave. The transmission electron microscopy (TEM) image in Fig. 1a confirmed the synthesis of two-dimensional nanosheet structure, and its X-ray diffraction (XRD) pattern in Fig. 1b well matches with the CuO phase (PDF#45-0937). Subsequently, the as-prepared CuO NS underwent electrochemical reduction process in a typical CO_2_RR flow-cell electrolyzer (Supplementary Fig. 2) equipped with a gas-diffusion-electrode (GDE, 0.5 cm^2^), and in situ converted into ER-CuNS catalyst after reaction under galvanostatic mode (20 mA cm^−2^) for 60 min in 0.1 M K_2_SO_4_ electrolyte. The XRD pattern proves the transformation of CuO to metallic Cu after the electrochemical reduction process (Fig. 1b). Intriguingly, a large number of pores were observed on nanosheet, as indicated by the low-magnification TEM and high-angle annular dark-field scanning transmission electron microscopy (HAADF-STEM) images (Supplementary Figs. 3–4 and Fig. 1c). The static size distribution analysis gives the average pore size of about 14 nm (Fig. 1c inset). Notably, the formation of the pores is likely related with the oxygen depletion during electrochemical reduction under well-controlled cathodic potential. The detailed structural information of ER-CuNS was further analyzed by the atomic-resolution aberration-corrected HAADF-STEM. As shown in Fig. 1d, e, Cu (111) and (100) facets with lattice spacing of 0.21 nm and 0.18 nm could be clearly resolved, respectively. The brightness contrast and height profile evidently affirm the porous structure (Fig. 1d, e). In addition, X-ray photoelectron spectroscopy (XPS) was performed as well to inspect the chemical state of ER-CuNS. It is hinted by high-resolution Cu 2*p* spectrum in Fig. 1f that Cu mainly appears as metallic state, while small amount of oxidized state is also detected probably arising from surface oxidation of Cu after exposing to air. All these above analyses have demonstrated the successful fabrication of ER-CuNS featuring porous nanosheet structure. It is expected that those abundant pores can regulate the reaction microenvironment by affecting the local distribution of reactive/non-reactive species near the catalyst surface, which would ultimately modulate the catalytic CO_2_RR behaviors^15^.

### CO_2_RR performance in strongly acidic electrolyte

**Fig. 2:**
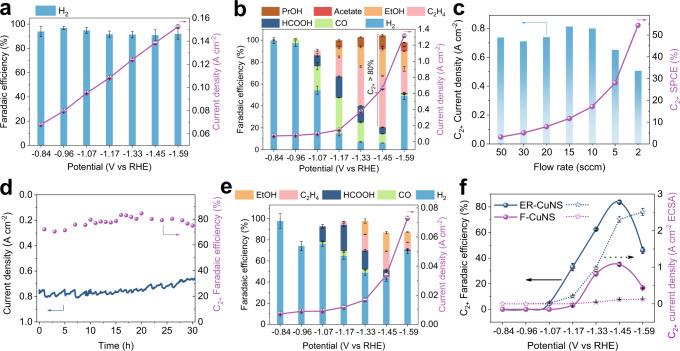
Electrocatalytic CO_2_RR performance in acidic electrolyte. **a** FE (left axis) and total current density (right axis) of ER-CuNS in 0.05 M H_2_SO_4_ at different applied voltages. **b** FE and total current density of ER-CuNS in 0.05 M H_2_SO_4_ with 3.0 M KCl additives. **c** Partial current density and SPCE of C_2+_ products on ER-CuNS at different CO_2_ gas flow rate with an applied voltage of −1.50 V_RHE_. **d** Total current density and C_2+_ FE of acidic CO_2_RR during long-term stability test on ER-CuNS at around −1.48 V_RHE_. The finally remaining C_2+_ FE is about 75.0%. **e** FE and total current density of F-CuNS in 0.05 M H_2_SO_4_ with 3.0 M KCl additives. **f** The C_2+_ FE and ECSA-normalized C_2+_ current density comparison between ER-CuNS and F-CuNS in 0.05 M H_2_SO_4_ with 3.0 M KCl additives. All FE and current density values are means with error bars (standard deviations) from three replicates.

Considering the advantages of acidic CO_2_RR in carbon utilization efficiency and long-term operation stability, we studied the CO_2_RR performance of as-synthesized ER-CuNS in strongly acidic electrolyte^18,21,26^. Preliminarily, Fig. 2a shows the product selectivity and total current density of ER-CuNS in pure 0.05 M H_2_SO_4_ at different potentials. Unfortunately, H_2_ is the exclusive product and no CO_2_RR products can be observed, in line with previous reports^19,21^. This demonstrates that kinetically favorable HER governs the current acidic system, and CO_2_ is not effectively activated. We found that the catalytic selectivity and activity for CO_2_RR in acidic electrolyte were substantially improved after introducing 3 M KCl into 0.05 M H_2_SO_4_ electrolyte (Fig. 2b and Supplementary Fig. 5). More importantly, the high FE of 83.7 ± 1.4% and the large partial current density of 0.56 ± 0.02 A cm^−2^ for C_2+_ products (including ethylene, ethanol, acetate acid, and n-propanol) were achieved at the potential of −1.45 V versus reversible hydrogen electrode (V_RHE_) in strong acid (pH ≤ 1). The SPCE towards C_2+_ production was also examined on ER-CuNS catalyst. Figure 2c plots the C_2+_ current density and SPCE against the CO_2_ gas flow rate. Due to the inhibited carbonate formation under acidic conditions, the C_2+_ current density can keep at a high level while the CO_2_ flow rate persistently declines. The highest SPCE of 54.4% for C_2+_ products is achieved at 2 sccm, surpassing the reported values in alkaline system, and demonstrating the advantage of acidic CO_2_RR^1,11,30–32^. Besides, we evaluated the full cell performance of acidic CO_2_RR by two-electrode measurement. It is found that a large cell voltage of −10 V is required to achieve the current density of 0.79 ± 0.04 A cm^−2^, where the high C_2+_ FE of 81.2 ± 1.9% can be realized (Supplementary Fig. 6). This CO_2_RR performance is very close to the results obtained by three-electrode measurement, verifying the good reproducibility of our catalytic system. It should be pointed out that such a high cell voltage should originate from the inappropriate design of our electrolyzer for full cell measurement, where the large system resistance is expected due to the thick cathode and anode chamber (15 mm) of our flow cell^21^. We also evaluated the long-term catalytic the stability of ER-CuNS toward acidic CO_2_RR in a three-electrode flow cell. As Fig. 2d indicates, no significant decay of current density and C_2+_ FE was observed after operation for 30 h, presenting the good electrolysis durability in acidic CO_2_RR field (Supplementary Table 2). The nanosheet structures of ER-CuNS were also basically remained after stability test (Supplementary Fig. 7). Taken these together, we herein actualized the efficient acidic CO_2_RR toward C_2+_ products on ER-CuNS, with high FE of 83.7 ± 1.4%, high C_2+_ current density of 0.56 ± 0.02 A cm^−2^, SPCE of 54.4% and stable electrolysis of 30 h, under the aid of K^+^ cation, representing a great step for acidic CO_2_ electrolysis (Supplementary Table 2).

For comparison, we also synthesized the flat Cu nanosheet (F-CuNS) catalyst through a one-step method (structure characterizations are shown in Supplementary Fig. 8). Figure 2e shows the CO_2_RR selectivity and activity of F-CuNS catalyst in 0.05 M H_2_SO_4_ with 3.0 M KCl additives. As shown in Fig. 2f, the highest FE of C_2+_ products on F-CuNS catalyst is only 35.1% at −1.45 V_RHE_, much inferior to 83.7% on ER-CuNS catalyst. To compare the intrinsic activity of these two catalysts, we calculated their electrochemical active surface areas (ECSA) through scanning cyclic voltammetry (CV) curves in the non-faradic region (Supplementary Figs. 9–10). By normalizing the C_2+_ partial current density against the ECSA, the specific activity was derived. Remarkably, the intrinsic activity of ER-CuNS catalyst for C_2+_ production (2.31 mA cm^−2^) is 22 times higher than that of F-CuNS catalyst (0.11 mA cm^−2^) at −1.45 V_RHE_ (Fig. 2f). Given the same electrolysis condition and similar nanosheet morphology, such great improvements in both selectivity and activity on ER-CuNS catalyst can be reasonably attributed to the presence of porous structure. Based on these above discussions, we can reasonably judge that the acidic CO_2_RR performance of ER-CuNS catalyst towards C_2+_ products originates from the K^+^ cation in acidic electrolyte and porous structure of the catalyst.

### Mechanistic understanding

**Fig. 3:**
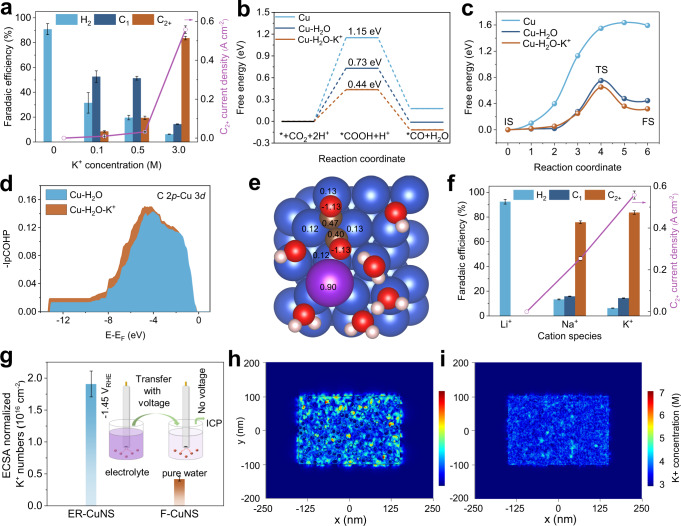
Mechanistic studies. **a** K^+^ concentration-dependent FE and C_2+_ current density of ER-CuNS at −1.45 V_RHE_. **b** Free energy diagrams of CO_2_RR-to-CO pathway on Cu (111) slab (Cu), Cu (111) slab with H_2_O (Cu–H_2_O), and Cu (111) slab with H_2_O and K^+^ species (Cu–H_2_O–K^+^) on the surface. **c** Reaction energy barriers of *CO dimerization on different slabs. IS, initial state. TS, transition state. FS, final state. **d** Integrated projected crystal orbital Hamilton population (-IpCOHP) curves of C 2*p*-Cu 3*d* bond for *OCCO intermediate adsorbed on Cu–H_2_O and Cu–H_2_O–K^+^ slab. **e** The charge density analysis of *OCCO on Cu–H_2_O–K^+^ slab according to the calculation of Bader charge. The blue, brown, red, white, and purple balls represent Cu, C, O, H, and K atoms, respectively. **f** CO_2_RR FE and C_2+_ current density of ER-CuNS in 0.05 M H_2_SO_4_ with 3 M alkali metal cations at −1.45 V_RHE_. **g** ECSA-normalized K^+^ number on F-CuNS and ER-CuNS. The inset is the operation method. **h**, **i** K^+^ distribution on ER-CuNS (**h**) and F-CuNS (**i**) models obtained from COMSOL Multiphysics finite-element-based simulations. All FE, current density, and K^+^ numbers values are means with error bars (standard deviations) from three replicates.

The underlying mechanism for the roles of K^+^ cation and porous structure in promoting CO_2_RR for C_2+_ production was further investigated in detail. For K^+^ cation, K^+^ concentration-dependent CO_2_RR performance in acidic medium was plotted, as shown in Supplementary Figs. 13–14 and Fig. 3a. With the increase of K^+^ concentration in acidic electrolyte, the FE of H_2_ gradually decreases and reaches as low as 6.3% at 3 M K^+^ (Fig. 3a). Meanwhile, the partial current density of H_2_ first decreases and then varies little after introducing more K^+^ (Supplementary Fig. 15). In terms of carbon-based products, the small amount of K^+^ (0.1 M) could bring about the significant improvement in the selectivity of C_1_ products especially CO. More K^+^ (0.5–3 M) is found to favor the production of C_2+_ products in terms of both selectivity and productivity accompanying with the decrease in C_1_ selectivity (Fig. 3a). The trade-off relationship was reported in previous studies, which can be ascribed to the electrochemical conversion of CO to C_2+_ products^33,34^. Overall, these experimental results clearly suggest that the K^+^ cation can suppress the competing HER and promote C_2+_ production simultaneously over ER-CuNS catalyst in strongly acidic electrolyte.

To shed light on how K^+^ cation suppresses competing the HER process, we studied the K^+^ concentration dependence of HER kinetics on ER-CuNS catalyst. From the linear sweep voltammetry (LSV) curves, the slower HER kinetics was evidenced at a higher K^+^ concentration (Supplementary Figs. 16–17). Meanwhile, a characteristic peak in LSV associated with proton diffusion limitation could be observed in the presence of K^+^ cation, which is confirmed by the rotation speed-dependent HER current density (Supplementary Figs. 18–21). This evidence suggests that the added K^+^ cation could decrease the HER kinetics by slowing the mass transport of proton. The quantitative effect of K^+^ concentration on the mass transport was further revealed by plotting the (j_HER_)^−1^ *vs* ω^−1/2^ curves for different K^+^ concentrations (Supplementary Fig. 22)^35^. The larger slope for higher K^+^ concentration corroborates the function of K^+^ cation on hindering mass transport of proton. About the picture of how K^+^ cation suppresses the mass transport, we could reasonably assume that the added K^+^ cation in acid can occupy the Helmholtz plane and lower the proton coverage via shielding the negative charge at the cathode surface (competitive adsorption effect)^12,24^. It is worth pointing out that K^+^ cations could have different effects on HER in alkaline media, where the K^+^ cations may facilitate the water dissociation via non-covalent interaction and accelerate the alkaline HER^36^.

Beside from the abovementioned kinetic aspect, the effects of adsorbed K^+^ cation on the energy profiles for CO_2_RR and HER were further investigated by the density functional theory (DFT) calculations from the perspective of thermodynamics. Prior to the calculations, the Cu (111) slab composed of four layers of (4 × 3 × 3) supercell with one K^+^ hydrated with six H_2_O molecules on the surface was modeled. The pure Cu (111) slab and slab with only H_2_O molecules on the surface were taken as references (Supplementary Fig. 23). The optimized configurations of key intermediates for CO_2_RR adsorbed on slabs are presented in Supplementary Figs. 24–26. Figure 3b depicts the free energy diagram of the CO_2_-to-CO pathway, where the formation of *COOH intermediate after the electron-proton transfer step acts as the rate-determining step (RDS). The free energies for the *COOH intermediate were calculated to be 1.15 eV, 0.73 eV, and 0.44 eV on Cu slab (Cu), Cu slab with H_2_O (Cu–H_2_O), and Cu slab with hydrated K^+^ (Cu–H_2_O–K^+^), respectively. It is thus concluded that K^+^ cation can significantly promote CO_2_ activation and *CO formation through strengthening adsorption of COOH* intermediate on Cu surface. Further analysis on the charge density indicates that the K^+^ increases the total electron density of *COOH intermediate, which leads to the stabilization of *COOH intermediate on the catalyst surface (Supplementary Fig. 27)^37^. In addition, the increased electron density when K^+^ exists can also stabilize the key *CO intermediate, in favor of its further conversion instead of direct desorption to CO (Supplementary Fig. 28).

The energy profiles of C–C coupling (*CO dimerization), the typical RDS for multicarbon production^5^, were also calculated on different slabs (Supplementary Figs. 29–31). The result shows that the *CO dimerization on Cu surface with hydrated K^+^ holds the lowest energy barrier (0.65 eV) in contrast to that on pure Cu (1.64 eV) or Cu–H_2_O (0.75 eV) surface (Fig. 3c), demonstrating the K^+^ cation can facilitate C–C coupling kinetics. To disclose the underlying mechanism, we then inspected the projected density of states (pDOS) and projected crystal orbital Hamilton populations (pCOHP) for the Cu-C bond of *OCCO after the C–C coupling step (Supplementary Fig. 32)^38^. As we can see, the C *2p* band and Cu 3*d* band overlap more on Cu–H_2_O–K^+^ slab. More specifically, the stronger bonding between *OCCO and Cu is further supported by the integrated projected crystal orbital Hamilton population (-IpCOHP) curves in Fig. 3d, where higher -IpCOHP value can be clearly observed at a wide energy range when K^+^ exists^39^. Further charge density analyses derived from the calculated Bader charge data demonstrate that K^+^ can locally interact with *OCCO through electron transfer process to increase the charge density of the intermediate (Fig. 3e and Supplementary Fig. 33), therefore stabilizing it and ultimately lowering the energy barrier of C–C coupling RDS. Besides, we also calculated the hydrogen adsorption energy to probe the effect of K^+^ cation on HER from the perspective of thermodynamics (Supplementary Fig. 34). Different from the case on the CO_2_RR pathway, the adsorbed K^+^ cation shows negligible impact on hydrogen adsorption energy, ruling out the possible thermodynamic effect for HER. Taking all these together, we revealed the K^+^ cation effect on suppressing the HER and accelerating the CO_2_RR to multicarbon products via modifying the microenvironment near ER-CuNS catalyst surface. Inspired by these analyses, we have also evaluated the effects of other alkali metal cations on acidic CO_2_RR, where Li^+^ fails to produce any carbon-based products, while Na^+^ achieves considerable C_2+_ FE of 75.8% and partial current density of 0.24 A cm^−2^ at similar conditions (Fig. 3f and Supplementary Fig. 35). The results prove the feasibility of alkaline metal cations especially with relatively large size, to boost the acidic CO_2_RR system.

We further attempted to understand the role of porous structure in improving the acidic CO_2_RR performance of ER-CuNS catalyst. Compared with the flat surface, it has been widely accepted that the porous structure would produce a distinct microenvironment near the catalyst surface via the confinement effect to alter the local distribution of reactive/non-reactive species^40,41^. Given the significant impact of K^+^ cation on CO_2_RR performance, we compared the amounts of K^+^ accumulated on ER-CuNS and F-CuNS under CO_2_RR conditions. Experimentally, the electrode loaded with ER-CuNS catalyst or F-CuNS catalyst was rapidly transferred from electrolyte to pure water while keeping voltage, and voltage was later shut to release K^+^ for inductively coupled plasma (ICP) analysis (Fig. 3g)^37^. The result shows that the value of ECSA-normalized K^+^ concentration on ER-CuNS is 4.5 times higher than that on F-CuNS, experimentally evidencing that the confinement effect from porous structure could concentrate K^+^ cation. To theoretically elucidate the confinement effect, COMSOL Multiphysics finite-element-based simulations were also conducted for ER-CuNS and F-CuNS. In accordance with the experimental result, the higher concentration of K^+^ cation is confined within the ER-CuNS, which can be ascribed to the amplified electric field near to pore sites in essence (Fig. 3h–i and Supplementary Fig. 36)^33,42^. Besides, the higher local alkalinity could be created in the porous channels according to the studies by Koper and co-workers, which also results in the rise of near-surface cation concentration^43,44^. These self-consistent studies thus demonstrate that the porous ER-CuNS catalyst would enrich K^+^ cation on the catalyst surface via confinement effect, which could reasonably account for the further improvements in activity and selectivity towards C_2+_ products.

**Fig. 4:**
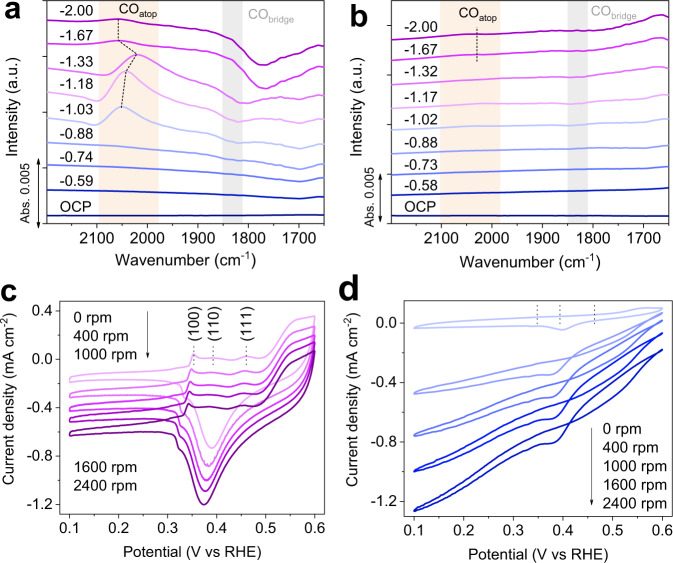
In situ experimental evidence for microenvironment modulation. **a**, **b** ECSA-normalized in situ ATR-SEIRAS spectra of acidic CO_2_RR collected on ER-CuNS (**a**) and F-CuNS (**b**) catalysts under different applied potentials (V_RHE_) in 0.05 M H_2_SO_4_ electrolyte with 3 M KCl additives. **c**, **d** ECSA-normalized rotation speed-dependent CV curves for OH^−^ adsorption on ER-CuNS (**c**) and F-CuNS (**d**) in N_2_-saturated 0.1 M KOH aqueous solution.

The optimized local microenvironment for the enhanced C_2+_ production on ER-CuNS was further examined by in situ techniques. It has been widely accepted that *CO functions as the critical role to form C_2+_ products through C–C coupling reaction^5^. Supplementary Fig. 37 presents the in situ attenuated total reflection surface-enhanced infrared absorption spectra (ATR-SEIRAS) of ER-CuNS in pure 0.05 M H_2_SO_4_ electrolyte during acidic CO_2_RR, where no signals attributing to *CO can be observed under all applied potentials, in consistence with the observation by Koper and co-workers^19^. This also echoes with the CO_2_RR performance in the pure acidic electrolyte that no carbon-based products is formed (Fig. 2a). As a sharp contrast, when 3 M K^+^ is introduced to boost the acidic CO_2_RR, the distinct peak belonging to *CO intermediate around 2050 cm^−1^ appears on ER-CuNS at a potential of −1.03 V_RHE_ (Fig. 4a)^45,46^. Further increasing overpotential leads to the reduced *CO coverage (after −1.18 V_RHE_) because of the consumption by C–C coupling reaction^33^. It should be mentioned that here the observed band mainly corresponds to *CO on atop sites (CO_atop_) that is more reactive for transformation into C_2+_ products, while the signal of relatively unreactive *CO on bridge sites (CO_bridge_) is nearly negligible^47^. In contrast, the CO_atop_ signal is much weaker on F-CuNS and only inconspicuously appears at very negative potentials (Fig. 4b). Such a much higher surface coverage of *CO intermediate on ER-CuNS can be ascribed to the promotional role of concentrated K^+^ cation in favoring the *CO formation. Besides, the confinement effect of porous structure may also account for the accumulated *CO, which in turn facilitates the C–C coupling for C_2+_ production^48^.

It was reported that OH^−^ species located at the catalyst surface could also benefit C–C coupling and C_2+_ production^11,49^. To verify the accumulation of OH^−^ species on ER-CuNS, cyclic voltammetry (CV) technique was employed, which detected the OH^−^ adsorption (OH_ad_) feature in situ. Figure 4c demonstrates the pronounced OH_ad_ peaks associated with Cu (100), (110), and (111) facets on ER-CuNS, respectively^50,51^. Noted that there is a current shifting with increasing the rotating speed probably related to the presence of cathodic oxygen reduction reaction given O_2_ cannot be totally removed in our measurements, but the current shifting could not impact our analysis on the OH_ad_ features. In particular, with the increase of rotation speed, the OH_ad_ peaks show negligible decay. As a comparison, the OH_ad_ peaks are very weak at 0 rpm on F-CuNS, and quickly disappear with increasing the rotation speed (Fig. 4d). Such a big difference in adsorption behavior verifies the confinement effect on accumulating the OH^−^ by the porous structure, which is believed as another promotional factor to expedite C_2+_ formation. In the meantime, the more enriched OH^−^ in porous structure is also beneficial to maintain a local alkaline environment to suppress the parasitic proton reduction. Besides, we also estimated the local pH of CO_2_RR at the electrode-electrolyte interface by using in situ Raman spectroscopy to monitor the generated HCO_3_^−^ or CO_3_^2−^ species under working conditions (Supplementary Figs. 38–39)^52^. According to the calculations based on equilibrium between HCO_3_^−^ and CO_3_^2−^ (Supplementary Note 1, Supplementary Fig. 40, Supplementary Tables 3–4), it is revealed that ER-CuNS exhibits higher local pH than F-CuNS at the same potentials. In this case, more OH^−^ species can be created and accumulated on reaction interface of ER-CuNS electrode to elevate the C_2+_ efficiency, in line with analysis from Fig. 4c, d. Combined all our analyses together, the acidic CO_2_RR performance on ER-CuNS catalyst can be convincingly ascribed to the advantageous synergies between cation effect and confinement effect in optimizing the microenvironment near the catalyst surface, which greatly reduce the proton coverage to suppress competing HER and promote the C–C coupling process. Of note, such synergistic modulation strategy enables the comparable C_2+_ FE performance for acidic CO_2_RR and alkaline CO_2_RR (Supplementary Fig. 6), highlighting the prospects of microenvironment engineering.

## Discussion

In summary, we have demonstrated a highly selective, efficient, and stable CO_2_RR for C_2+_ production over ER-CuNS catalyst in acidic electrolyte via combining cation effect and confinement effect to modulate the microenvironment over the catalyst surface. A high FE of 83.7 ± 1.4%, large partial current density of 0.56 ± 0.02 A cm^−2^, high SPCE of 54.4%, and stable electrolysis of 30 h, were achieved for C_2+_ products in strong acid (pH ≤ 1). The mechanistic studies have comprehensively provided insights into how cation effect and confinement effect boost C_2+_ production. On the one hand, the presence of K^+^ cation in electrolyte would kinetically reduce the proton coverage on the Helmholtz plane through the competitive adsorption behavior driven by the electrostatic field and thermodynamically favor the *CO production and C–C coupling via stabilizing the key intermediates. On the other hand, the confinement effect arising from the porous structure of ER-CuNS catalyst would concentrate the K^+^ cation and OH^−^ species near the catalyst surface, both of which could promote the CO_2_RR to C_2+_ products. Beyond a promising catalytic system for efficient CO_2_RR, our findings also provide a general guidance to steer the electrochemical process by tailoring the reaction interface.

## Methods

### Characterizations

The morphology and structure of ER-CuNS and F-CuNS were investigated using transmission electron microscopy (JEOL JEM-2100Plus) and the high-angle annular dark-field scanning transmission electron microscopy with a spherical aberration corrector (HAADF-STEM, Thermo scientific Themis Z 3.2). The crystalline phases of all samples were evaluated by X-ray diffraction (XRD, Rigaku Miniflex-600) with a Cu Kα radiation (λ = 0.15406 nm, 40 kV). X-ray photoelectron spectroscopy (XPS) spectrum was collected by using a Thermo Scientific K-Alpha spectrometer equipped with an Al Kα (hv = 1486.6 eV) excitation source.

### Synthesis of electrochemically reduced Cu nanosheet (ER-CuNS)

Typically, 10 mL of 6 M NaOH aqueous solution was added dropwise to 10 mL of 100 mM CuCl_2_ aqueous solution under magnetic stirring (1000 rpm). The mixture was kept stirring at room temperature for 30 min. After that, the mixture was transferred into a Teflon-lined autoclave, capped, and heated at 100 °C for 12 h. After cooling down to room temperature, the resulting product was collected by centrifugation. The product was washed several times with ultrapure water as well as ethanol, and then dried in a vacuum oven at 50 °C overnight. Subsequently, the ER-CuNS true catalyst was obtained via in situ electrochemical reduction from CuO NS loaded on gas-diffusion-electrode (GDE) embedded within flow cell electrolyzer (detail see section “Preparation of working electrode” below).

### Synthesis of flat Cu nanosheet (F-CuNS)

The F-CuNS were synthesized according to a reported method^53^. Typically, Cu(NO_3_)_2_·3H_2_O (50 mg) and L-ascorbic acid (100 mg) were mixed with 15 mL of ultrapure water, and the mixture was kept stirring at room temperature for 30 min to form a homogeneous solution. Then hexadecyl trimethyl ammonium bromide (100 mg) and hexamethylenetetramine (100 mg) were added followed by 30 min of stirring. The mixture solution was purged with N_2_ for 15 min to remove the trapped O_2_. After that, the vial was transferred into an oil bath set to 80 °C. The reaction was continued under magnetic stirring for 3 h. The resulting product was collected by centrifugation. The product was washed several times with ultrapure water as well as ethanol, and finally dispersed in ethanol for use.

### Preparation of working electrode

(1) *ER-CuNS electrode*. Typically, 14 mg of CuO NS and 7 mL of ethanol were mixed by sonicating for 60 min, and 30 μL of Nafion solution was added, followed by sonicating for another 60 min to obtain a homogeneous catalyst ink. The catalyst ink was then sprayed on hydrophobic porous polytetrafluoroethylene GDE (2 cm × 2 cm). The GDE before and after loading catalysts was weighed to determine the loading amount of the catalyst (1.7 mg cm^−2^). The ER-CuNS electrode was obtained via in situ electrochemical reduction from CuO NS electrode under galvanostatic mode for 60 min in 0.1 M K_2_SO_4_, with constant current density at 20 mA cm^−2^. (2) *F-CuNS electrode*. The 14 mg as-prepared F-CuNS sample was dispersed in 7 mL ethanol and ultrasonicated for 60 min. Later, 30 μL of Nafion solution was added, followed by sonicating for another 60 min to obtain a homogeneous catalyst ink. The catalyst ink was sprayed on hydrophobic GDE, with a loading amount of F-CuNS catalyst at 1.7 mg cm^−2^ as well.

### Preparation of IrO_x_/Ti-mesh anode

The IrO_x_/Ti-mesh anode was prepared via a dip coating and thermal deposition method^54^. Typically, Ti-mesh was first washed with ultrapure water and acetone, and then etched for 45 min in 6 M HCl at 80–90 °C. After that, the dip coating solution was obtained by dissolving 30 mg IrCl_3_·xH_2_O into 10 mL isopropanol with 10 v% HCl. Subsequently, the etched Ti-mesh was dipped into the dip coating solution, then dried for 10 min at 100 °C in the oven and underwent calcination at 500 °C for 10 min in a muffle furnace under air atmosphere. The dipping and calcination procedure was repeated for several times until the IrO_x_ loading reached about 2 mg cm^−2^. Finally, the resulting IrO_x_/Ti-mesh anode was used for subsequent electrochemical acidic CO_2_RR measurements.

### Electrochemical measurements

The acidic CO_2_RR performance was evaluated in a three-electrode system in a flow cell assembly (Supplementary Fig. 2). The used flow cell assembly consists of gas flow chamber, anolyte chamber, and catholyte chamber. Each chamber contained an inlet and outlet for gas or electrolyte. The window of the electrode exposed was a square with an area of 0.5 cm^2^. The anolyte chamber was separated from the catholyte chamber by a Nafion 117 cation exchange membrane (DuPont). 25 mL of 0.05 M H_2_SO_4_ containing various contents of KCl (0/0.1/0.5/3 M, corresponding to electrolyte pH of 0.97/0.90/0.83/0.51) was used as catholyte, and 25 mL of 0.05 M H_2_SO_4_ aqueous solution was used as anolyte. The electrolyte in cathode and anode were circulated by a peristaltic pump. During the measurements, high-purity CO_2_ (99.999%) gas was continuously supplied to the gas chamber at a rate of 50 sccm (or 2/5/10/15/20/30 sccm). The as-obtained ER-CuNS electrode or F-CuNS electrode was utilized as the working electrode. The Ag/AgCl (3.5 M KCl) and IrO_x_/Ti-mesh were employed as the reference electrode and counter electrode, respectively. All potentials were measured against an Ag/AgCl reference electrode, and converted to the reversible hydrogen electrode (RHE) reference scale via Nernst function with iR compensation as below:

1 $${{\mbox{E(V vs RHE)=E(V vs Ag/AgCl)+}}}0{{\mbox{.}}}208{{\mbox{+}}}0{{\mbox{.}}}0591\times {{\mbox{pH+}}}0{{\mbox{.}}}85\times {{\mbox{iR}}}$$

All the electrochemical tests were conducted in a three-electrode system using a DH7001A electrochemical workstation (Donghua Testing Technology Co., Ltd.), at room temperature. The electrochemical impedance spectroscopy (EIS) study was investigated by applying an open circuit voltage in a frequency range from 100 kHz to 0.1 Hz with an amplitude of 5 mV (Supplementary Figs. 11–12, Supplementary Table 1). The linear sweep voltammetry (LSV) experiments were scanned in acidic electrolyte with the scan rate of 50 mV s^−1^. Electrochemical active surface area (ECSA) of ER-CuNS and F-CuNS was determined by scanning cyclic voltammetry (CV) curves at non-faradaic region (0.05–0.07 V_RHE_) at varying scan rates (1–15 mV s^−1^). The OH^−^ adsorption curves of ER-CuNS and F-CuNS were tested through CV method at a scan rate of 50 mV s^−1^ in 1 M KOH^33^. Acidic CO_2_RR measurements were conducted under potentiostatic model, while gas products and liquid products were determined severally. The stability measurements of CO_2_RR under acid media were performed at potentiostatic conditions (−1.45 V_RHE_) to record the current density and FE of ER-CuNS in 0.05 M H_2_SO_4_ and 3 M KCl catholyte within 30 h.

### CO_2_RR product analysis

Unless otherwise stated, CO_2_ gas was led into gas chamber of flow cell at ambient pressure and room temperature, and then injected into a gas chromatograph (GC, Panna A60) after CO_2_RR to analyze gas products. The GC was equipped with a thermal conductivity detector (TCD) for analyzing H_2_, and a flame ionization detector (FID) for analyzing carbonaceous substances, while calibrated by using standard gas (Dalian special gases CO., LTD) before measurements. Each quantitative sampling was performed three times to achieve accurate results. The FE of gas products was calculated as follows:

2 $${{{{\rm{FE}}}}}\left(\%\right)=\frac{{{{{{\rm{Q}}}}}}_{{{{{\rm{gas}}}}}}}{{{{{{\rm{Q}}}}}}_{{{{{\rm{total}}}}}}} \times 100\%=\frac{NFvcP}{60 \times {JRT}} \times 100\%$$

where *N* is the number of transferred electron for targeted products, Faraday constant *F* = 96,485 C mol^−1^, *v* is the gas flow rate measured by a flow meter, *c* is the volume concentration of gas products (CO, CH_4_, C_2_H_4_, or H_2_) from the GC data, pressure *P* = 1.01 × 10^5 ^Pa, gas constant *R* = 8.314 J mol^−1^ K^−1^, temperature *T* = 298.15 K, *J* means the total recorded current.

On the other hand, liquid products were diluted and analyzed by ^1^H NMR (Bruker AVANCE III HD 400 MHz) with water peak suppression, in which 100 μL of the catholyte was prepared with 10 μL dimethyl sulfoxide (DMSO, 1200 ppm, the internal standard solution), 90 μL D_2_O and 400 μL H_2_O. The concentrations of liquid products were elucidated by its NMR peak area relative to the internal standard. FE of liquid products was determined as below:

3 $${{{{\rm{FE}}}}}\left(\%\right)=\frac{{{{{{\rm{Q}}}}}}_{{{{{\rm{liquid}}}}}}}{{{{{{\rm{Q}}}}}}_{{{{{\rm{total}}}}}}} \times 100\%=\frac{nNF}{Jt} \times 100\%$$

where *n* is the moles of liquid product in the cathodic compartment, *N* is the electron transfer number, *F* = 96,485 C mol^−1^, *t* is the reaction time, *J* is the recorded current. The partial current density under different applied potentials was determined by multiplying corresponding FE of each component and the total geometric current density. Note that for every set of data, three individual repeated measurements using the same batch of prepared electrodes were conducted to obtain the average FE and current density values with corresponding error bars (standard deviations).

The SPCE of CO_2_ towards producing C_2+_ was calculated as follows at 25 °C, 1 atm:

4 $${{{{{\rm{SPCE}}}}}}=	 \left(j\times 60\,{{{{{\rm{s}}}}}} \right)/\left(N\times F\right)\div\big({{{{{\rm{flow}}}}}}\,{{{{{\rm{rate}}}}}}\left({{{{{\rm{L}}}}}}/\,{{{{{\rm{min}}}}}}\right) \\ 	 \times 1\left({{{{{\rm{min }}}}}}\right)\big)/\left(24.05({{{{{\rm{L}}}}}}/\,{{{{{\rm{min }}}}}})\right)$$

where *j* means the partial current density of C_2+_, *N* stands for electron transfer^21^. Note that for precisely analyzing gas products and determining SPCE at very low CO_2_ gas flow rate (2 sccm), the GC standard curve was re-calibrated by using standard gas with higher concentration (tens of thousands ppm), and CO_2_RR operation time was extended to 4 h to let the system totally enter the steady state before collecting data.

### Hydrogen evolution reaction (HER) test

HER test was performed with a CHI660E workstation by using a three-electrode setup in a single cell, to evaluate the HER performance of ER-CuNS in different electrolytes (0.05 M H_2_SO_4_ with 0/0.5/1/2/3 M KCl aqueous solution). Glassy carbon rotating disk electrode (GC-RDE, 0.196 cm^2^) loaded with catalyst was used as the working electrode. The carbon rod and Ag/AgCl (3.5 M KCl) electrode were used as the counter electrode and reference electrode, respectively. Before measurements, the electrolytes were saturated by N_2_ for 10 min to remove O_2_ purity. LSV measurements were performed with a scan rate of 10 mV s^−1^ at different rotation speed.

### Measurement of electric-field-induced enrichment of K^+^

Electric-field-induced K^+^ enrichment was measured in an electrolyte similar to the catholyte for acidic CO_2_RR. The electrode loaded with ER-CuNS or F-CuNS was first conducted in 0.05 M H_2_SO_4_ aqueous solution with 3 M KCl additives at −1.45 V_RHE_ (without iR compensation). After running for 120 s, the electrode was directly raised above the electrolyte and transferred into 5 mL pure water, during which the voltage was kept. After immersing in water, the voltage was removed to release any adsorbed K^+^ from the electrode^37^. The transferred electrodes from the same aqueous solution without applying voltage were used as the blank background. Subsequently, the amount of K^+^ in the water was determined using an inductively coupled plasma optical emission spectrometer (ICP-OES, Atom scan Advantage, Thermo Jarrell Ash, USA). Finally, the amount of K^+^ in ultrapure water with the background deducted represents the true amount of K^+^ adsorbed on the surface of the ER-CuNS or F-CuNS catalysts. The obtained results were normalized by ECSA for comparison.

### In situ attenuated total reflection surface-enhanced infrared absorption spectroscopy (ATR-SEIRAS)

ATR-SEIRAS was carried out on a Nicolet iS50 FT-IR spectrometer equipped with an MCT detector cooled with liquid nitrogen. The Au-coated Si semi-cylindrical prism (20 mm in diameter) was used as the conductive substrate for catalysts and the IR reflection element. The catalysts suspensions were dropped on the Au/Si surface as the working electrode. The mass loading of the catalyst was 1 mg/cm^−2^ and the electrolyte was 0.05 M H_2_SO_4_ with/without 3 M KCl additives. In situ ATR-IR spectra were recorded during the stepping of the working electrode potential.

### In situ Raman spectroscopy

To determine the local pH on the electrode surface under CO_2_RR working conditions, in situ Raman spectra were acquired using a confocal Raman microscope (WITec Alpha 300R). The excitation source was a 633 nm laser with the power of 3 mW and grating of 600 grooves/mm, and a 50× objective (Zeiss LD EC Epiplan-Neofluar Dic) was used. Each spectrum was obtained with an acquisition time of 10 s and 3 times of accumulation. The electrochemical reactor for in situ Raman measurements was a C031-4 CO_2_RR flow cell purchased from Wuhan Gaoshi Ruilian Technology Co., Ltd, and ER-CuNS or F-CuNS catalyst was loaded on the carbon paper GDE and integrated into the flow cell. Details to calculate the local pH based on the Raman signal of HCO_3_^−^ and CO_3_^2−^ ions at the electrode surface can be found in Supplementary Information Note S1.

### DFT calculations

In this work, all calculations are carried out within the Perdew-Burke-Ernzerhof generalized gradient approximation (GGA)^55^ with D3 type van der Waals interaction (vdW) correction^56,57^ implemented in Vienna ab initio simulation package (VASP)^58^. The projector augmented wave (PAW) potential^59^ and the plane-wave cut-off energy of 450 eV are used. Our calculations have used a slab model composed of four layers of 4 × 3 × 3 representing the Cu (111) surfaces separated by 15 Å of vacuum space. The slabs and adsorbate configurations in this work include a single layer of H_2_O with and without a K^+^. Adsorbates and the top two layers of the slab were geometrically relaxed for each binding site, and the most stable adsorbate configuration was used to determine the electronic component of the free energy. The 3 × 3 × 1 Monk-horst k-point meshes were used for the Brillouin-zone integrations of supercell models. The criteria of convergence were set to 1 × 10^−5 ^eV for the self-consistent field (SCF) and 0.02 eV/Å for ion steps. We also employed the climbing image nudged elastic band method to determine the transition state for CO coupling on the Cu (111) surfaces^60^. Moreover, the force convergence tolerance on each atom was set to be 0.05 eV/Å.

### COMSOL multiphysics simulations

The electric field and K^+^ concentration within the vicinity of Cu electrodes were simulated by solving the Poisson-Nernst-Planck equations using the COMSOL Multiphysics finite-element-based solver (https://www.comsol.com/). The Nernst-Planck equations in the steady state used to solve the ion concentration distribution of solution species are given by:

5 $$\nabla \cdot \left({D}_{i}\nabla {c}_{i}+\frac{{D}_{i}{z}_{i}F}{RT}{c}_{i}\nabla \psi \right)=0$$

where *c*_*i*_, *D*_*i*_, and *z*_*i*_ are the concentration, the diffusion coefficient (*D*_1_ = 1.957 × 10^−9^ m^2^/s, *D*_2_ = 9.311 × 10^−9^ m^2^/s, *D*_3_ = 1.97 × 10^−9^ m^2^/s, and *D*_4_ = 1.065 × 10^−9^ m^2^/s)^61^, and the charge valence (*z*_1_ = *z*_2_ = +1, *z*_3_ = −1 and *z*_4_ = −2) of species *i* (1 for K^+^, 2 for H^+^, 3 for Cl^−^, and 4 for SO_4_^2−^), respectively. In addition, *F*, *R*, and *T* represent the Faraday constant, gas constant, and absolute temperature (*T* = 293.15 K), respectively, and *ψ* is the electrostatic potential that satisfies the Poisson equation:

6 $$\nabla \cdot \left({D}_{i}\nabla {c}_{i}+\frac{{D}_{i}{z}_{i}F}{RT}{c}_{i}\nabla \psi \right)=0$$

where *ε*_0_ is the permittivity of vacuum and *ε*_*r*_ is the relative permittivity of water (*ε*_*r*_ = 78). The electrical double layer (EDL) was modeled using the Gouy-Chapman-Stern model, which consists of a Helmholtz layer and a diffusion layer. The thickness of the Helmholtz layer was taken as the radius of a hydrated potassium ion (0.33 nm)^62^. The diffusion layer was established as the result of a dynamic equilibrium between electrostatic forces and diffusion. The so-called outer-Helmholtz plane (OHP) separates the EDL at the electrolyte side from the Helmholtz layer toward the bulk electrode side. Two three-dimensional models of 300 × 200 × 20 nm^3^ were built to represent the local porous structure and the smooth surface of Cu electrodes according to the TEM images, which were considered to be immersed into the electrolyte box of 500 × 400 × 220 nm^3^, as shown in Fig. 3h–i. In the system, the partial differential Eqs. (1)–(2) are solved under the following initial and boundary conditions. The initial values of species concentrations without applied potential were assumed to be same in the bulk electrolyte (3 M KCl and 0.05 M H_2_SO_4_). Meanwhile, Dirichlet boundary conditions specify the concentration of species and zero potential in the bulk. To ensure the accuracy of the theoretical model, a small electrode potential of 0.05 V was applied through all simulations. The electric field value and the potential value at the OHP were used as mixed boundary condition for the equation:

7 $$\nabla ({\varepsilon }_{r}{\varepsilon }_{0}\nabla \psi )=0$$

Free tetrahedral meshes were used for all simulations. Meshes were set to be the densest at the surface of the electrodes, where the element size was 0.2 nm.

## Supplementary information

Supplementary Information File

## Data Availability

All data needed to evaluate the conclusions in the paper are presented in the paper and/or the Supplementary Information. The data that support these findings are available from the corresponding author upon reasonable request. Source data are provided with this paper.

## Source data

Source Data
