# Supplementary material for: CO2 electroreduction to multicarbon products in strongly acidic electrolyte via synergistically modulating the local microenvironment
Source: Nat Commun. 2022 Dec 9;13:7596. doi: 10.1038/s41467-022-35415-x (PMC9734127; doi:10.1038/s41467-022-35415-x)
Supplement: Supplementary file 1 — Supplementary Information File [file 41467_2022_35415_MOESM1_ESM.pdf]

Supplementary Information

CO_2 electroreduction to multicarbon products in strongly acidic electrolyte via synergistically modulating the local microenvironment

Zesong Ma,[1,‖] Zhilong Yang,[1,‖] Wenchuan Lai,[1,‖,*] Qiyou Wang,[2] Yan Qiao,[1] Haolan Tao,[3] Cheng Lian,[3] Min Liu,[2] Chao Ma,[1] Anlian Pan[1] and Hongwen Huang[1,4,*]

[1]College of Materials Science and Engineering, Hunan University, Changsha, Hunan 410082, China

[2]State Key Laboratory of Powder Metallurgy, School of Physical and Electronics, Central South University, Changsha, Hunan 410083, China

[3]State Key Laboratory of Chemical Engineering and Shanghai Engineering Research Center of Hierarchical Nanomaterials, School of Chemistry and Molecular Engineering, East China University of Science and Technology, Shanghai 200237, China

[4]Shenzhen Research Institute of Hunan University, Shenzhen, Guangdong 518055, China

[‖] These authors contributed equally to this work.

Correspondence and requests for materials should be addressed to Wenchuan Lai (laiwenchuan@hnu.edu.cn) or Hongwen Huang (huanghw@hnu.edu.cn).

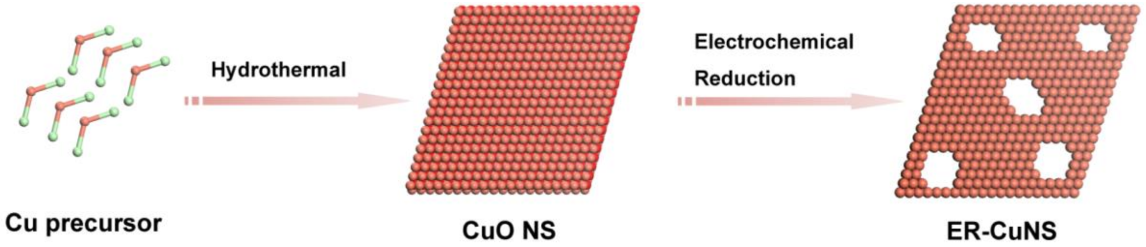

Supplementary Fig. 1 Preparation process of ER-CuNS comprised of hydrothermal and electrochemical reduction step.

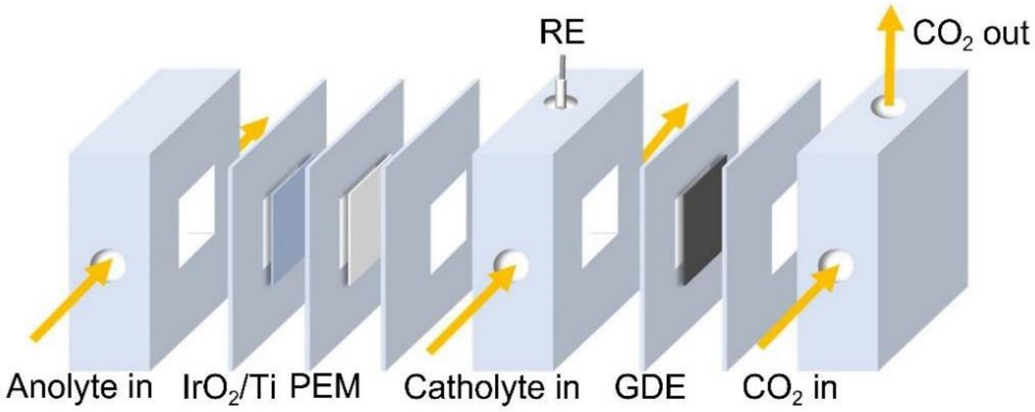

Supplementary Fig. 2 Schematic of flow cell reactor used for CO_2RR.

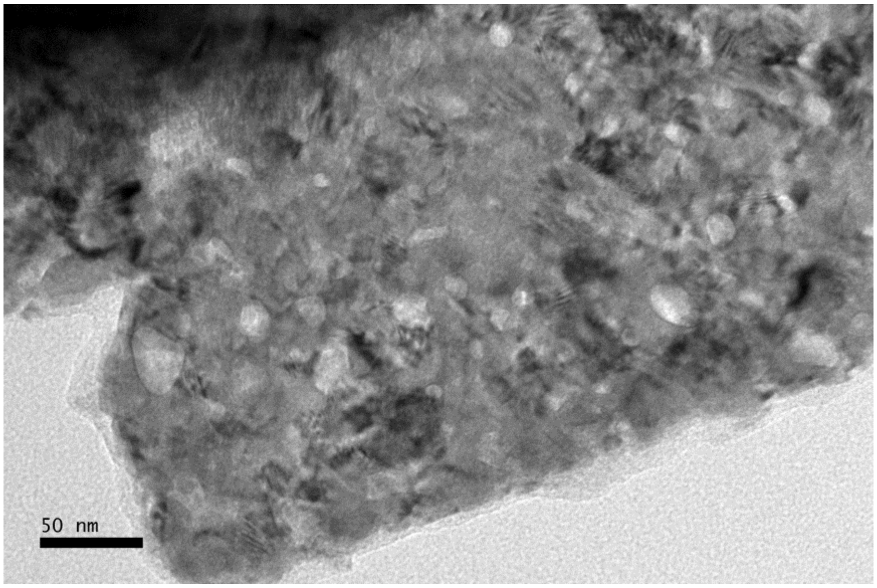

Supplementary Fig. 3 Low-magnification TEM image of ER-CuNS.

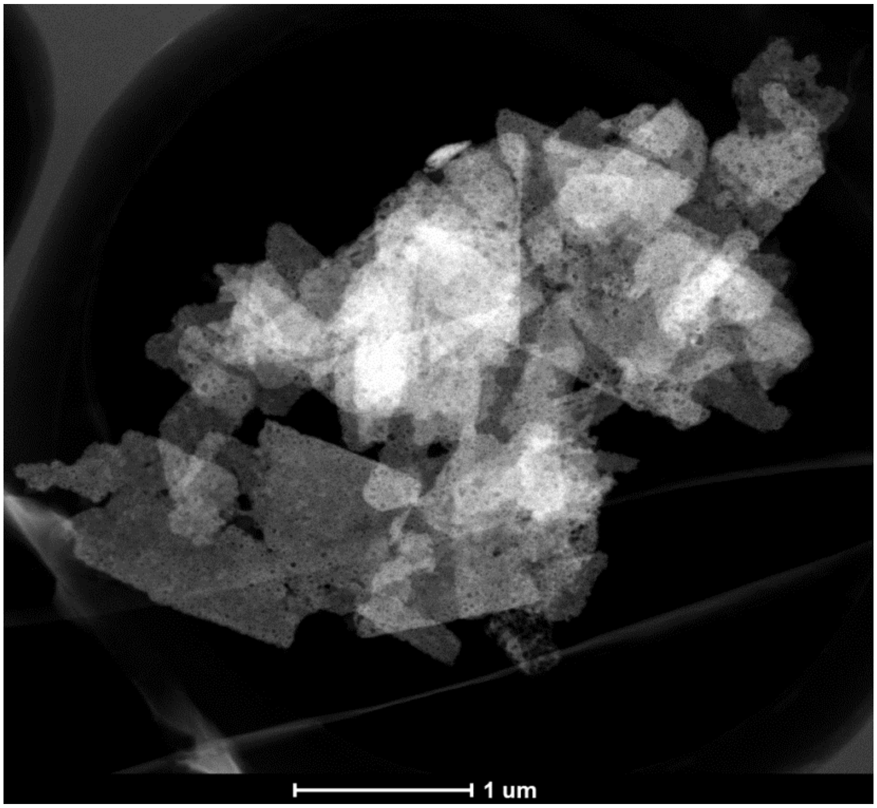

Supplementary Fig. 4 Low-magnification HADDF-STEM image of ER-CuNS.

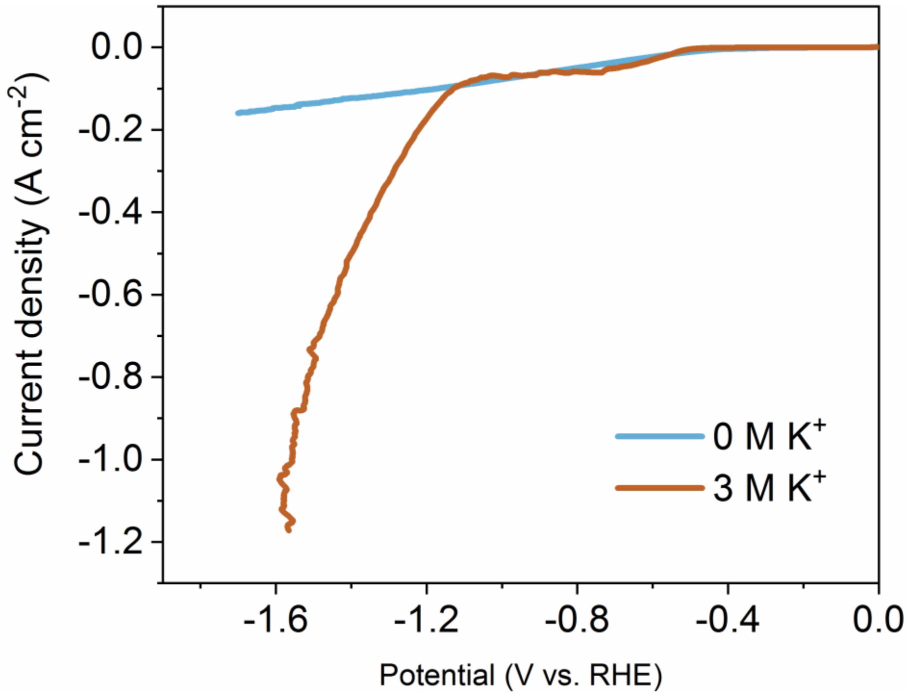

Supplementary Fig. 5 CO_2RR LSV curves of ER-CuNS in 0.05 M H_2SO_4 with 0 M or 3.0 M K^+ additives.

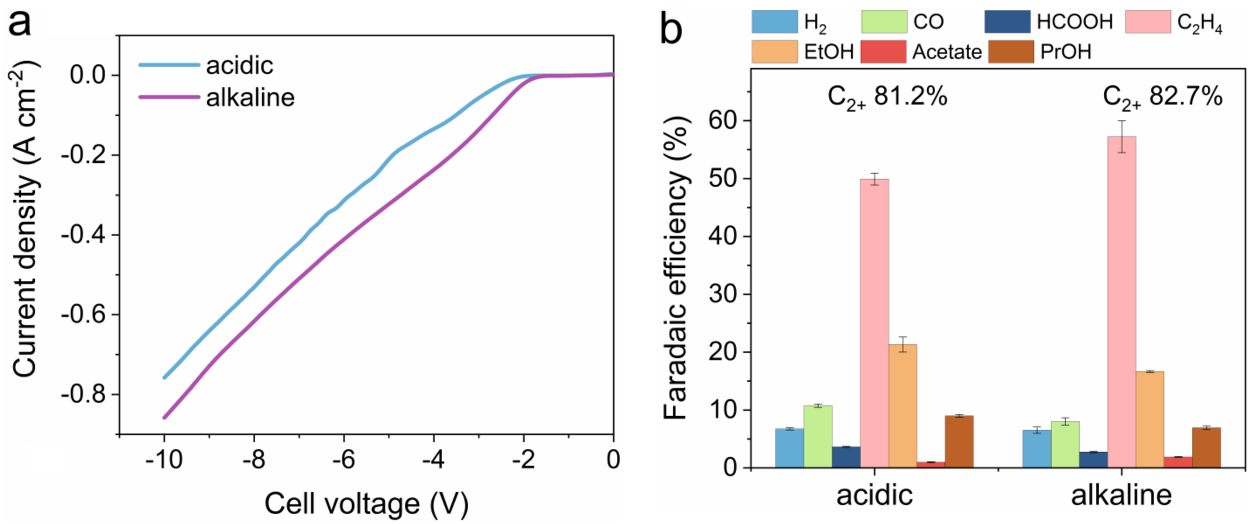

Supplementary Fig. 6 Comparison of full-cell CO_2RR performance between acidic and alkaline electrolyte. (a) LSV curves. For acidic system, the catholyte was 0.05 M H_2SO_4 + 3 M KCl and anolyte was 0.05 M H_2SO_4, using proton-exchange membrane (PEM) and IrO_2/Ti mesh as anode OER catalyst, with system resistance of 17.6 Ω. For alkaline system, the catholyte and anolyte were both 3 M KOH, using anion-exchange membrane (AEM) and IrO_2/Ti mesh as anode OER catalyst, with system resistance of 11.8 Ω. (b) Products distribution recorded from potentiostatic model (cell voltage, 10 V). The recording current density was about 0.79 $\pm$0.04 A cm^{-2} and 0.86 $\pm$0.02 A cm^{-2} for acidic and alkaline system, respectively.

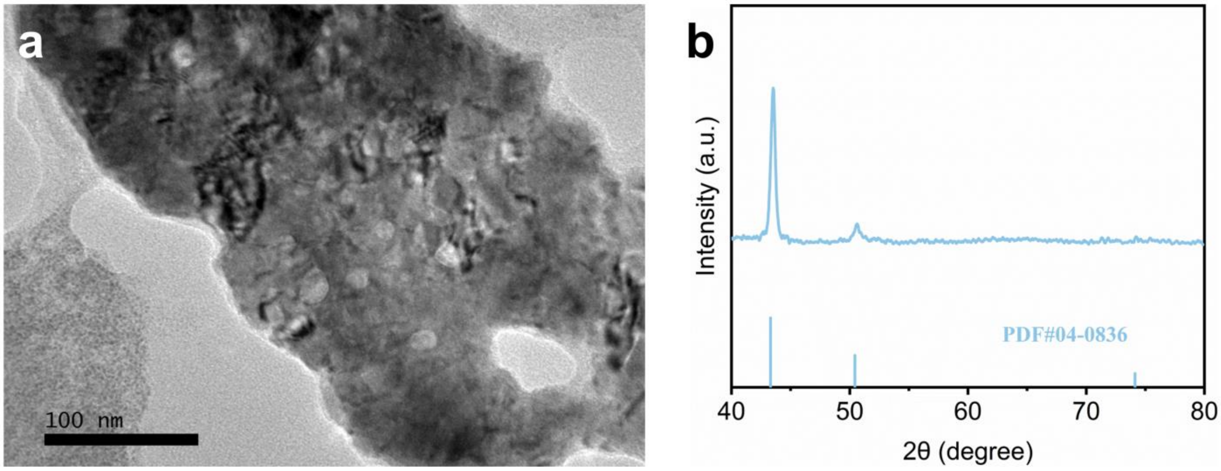

Supplementary Fig. 7 (a) TEM image and (b) XRD pattern of ER-CuNS after long-term durability test.

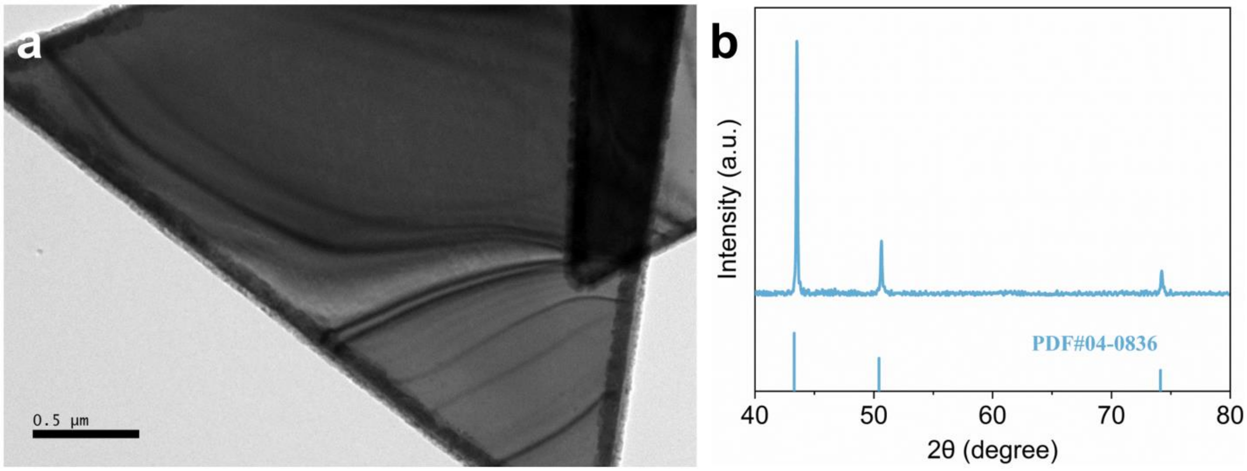

Supplementary Fig. 8 (a) TEM image and (b) XRD pattern of F-CuNS.

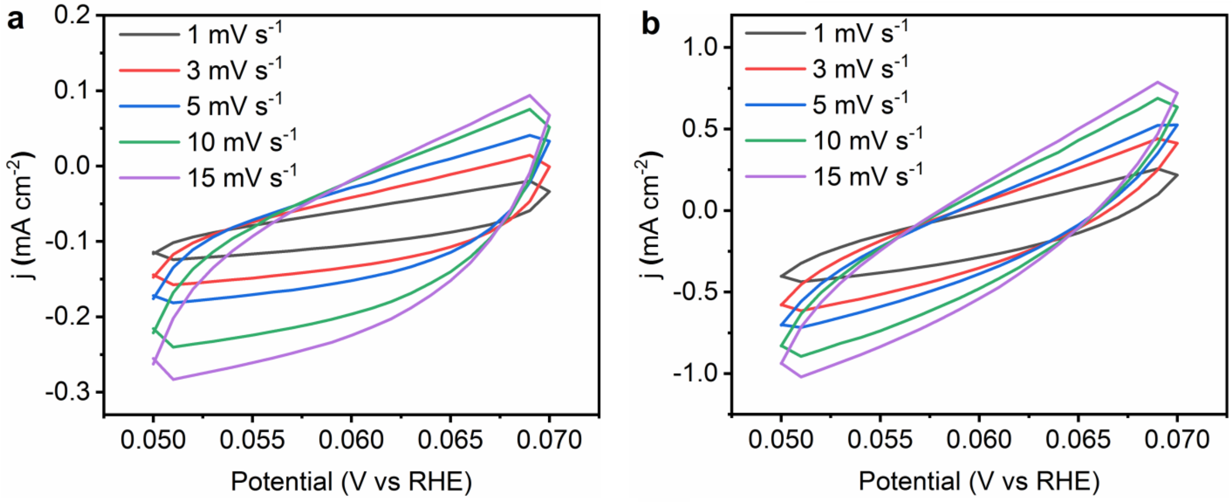

Supplementary Fig. 9 CV curves *vs* scan rate of (a) F-CuNS and (b) ER-CuNS in 0.05 M H_2SO_4 with 3.0 M KCl additives.

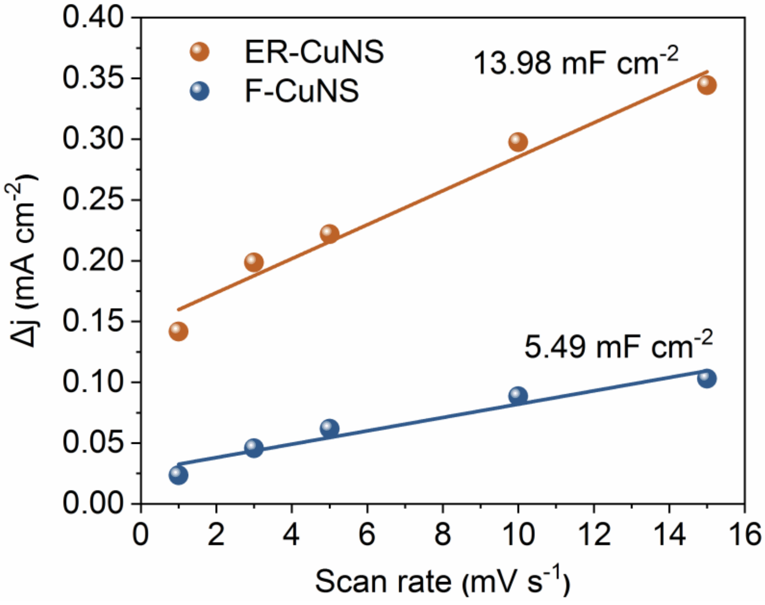

Supplementary Fig. 10 The double-layer capacitance of F-CuNS and ER-CuNS through fitting CV curves.

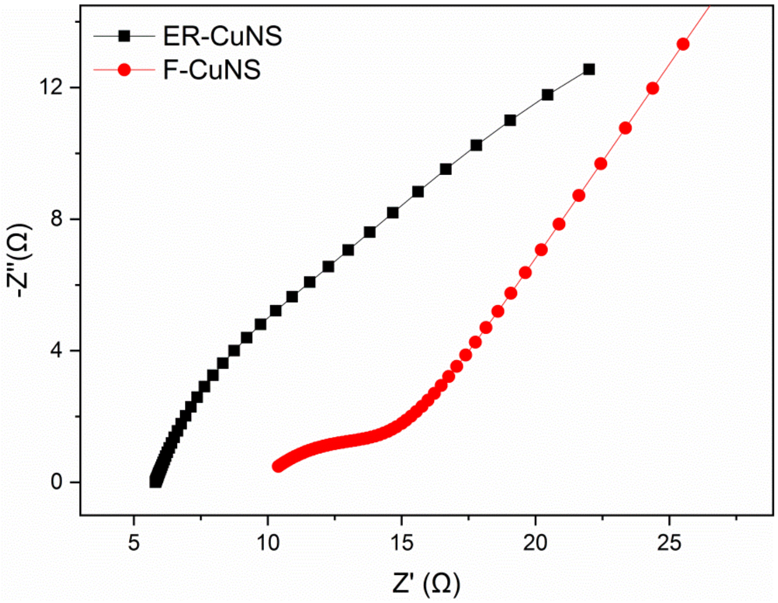

Supplementary Fig. 11 The fitted Nyquist plots of acid system of ER-CuNS and F-CuNS in 0.05 M H_2SO_4 electrolyte with 3 M KCl. All measurements were conducted at open circuit voltage status. The solution resistance of ER-CuNS and F-CuNS for compensating was 5.8 and 9.7 Ω, in acidic electrolyte with 3 M KCl. The fitted parameters of the EIS plots are displayed in Supplementary Table1.

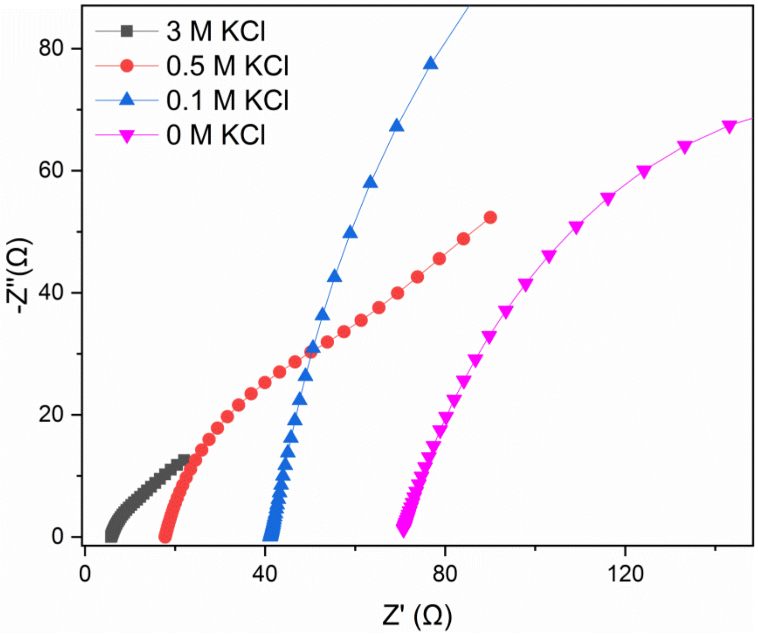

Supplementary Fig. 12 The fitted Nyquist plots of acid system of ER-CuNS in 0.05 M H_2SO_4 electrolyte with different concentration of KCl. All measurements were conducted at open circuit voltage status. The solution resistance for compensating iR was 5.8, 11.8, 41.1 and 70.1 Ω, respectively, corresponding to acidic electrolyte with 3 M, 0.5 M, 0.1 M and 0 M KCl additive. The fitted parameters of the EIS plots are displayed in Supplementary Table1.

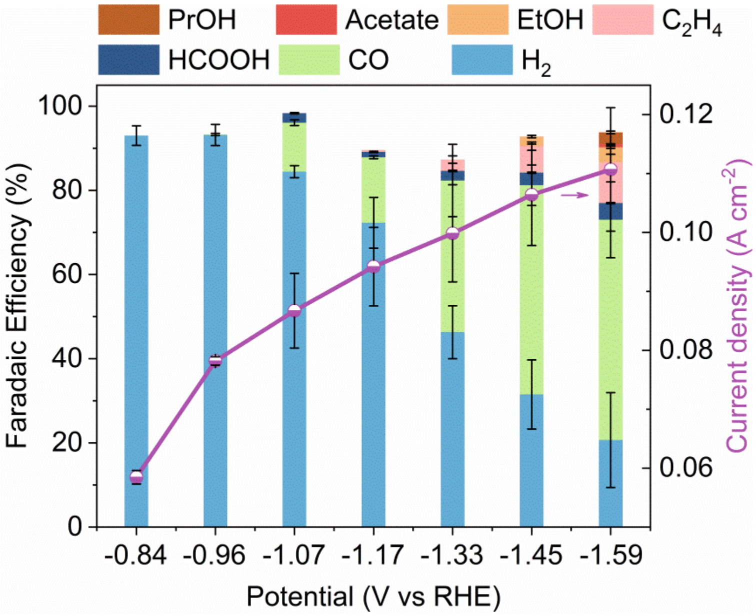

Supplementary Fig. 13 Faradaic efficiency (left axis) and total current density (right axis) of ER-CuNS in 0.05 M H_2SO_4 with 0.1 M KCl additives.

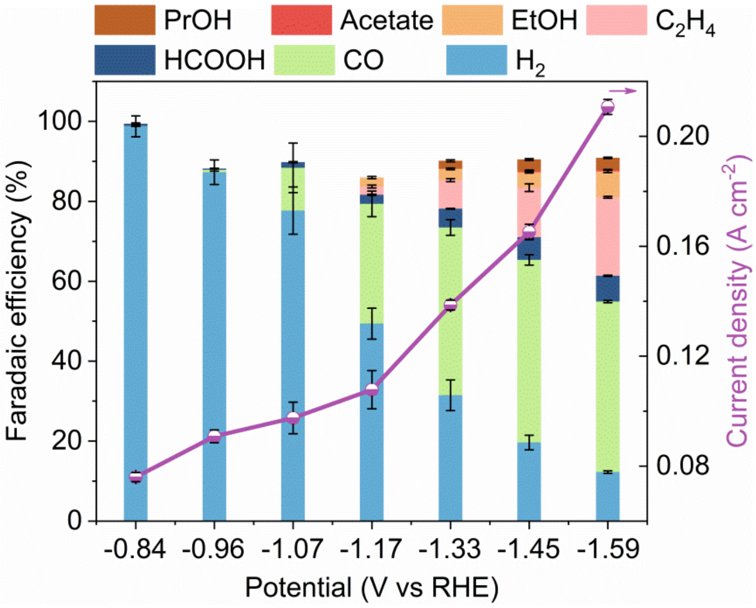

Supplementary Fig. 14 Faradaic efficiency (left axis) and total current density (right axis) of ER-CuNS in 0.05 M H_2SO_4 with 0.5 M KCl additives.

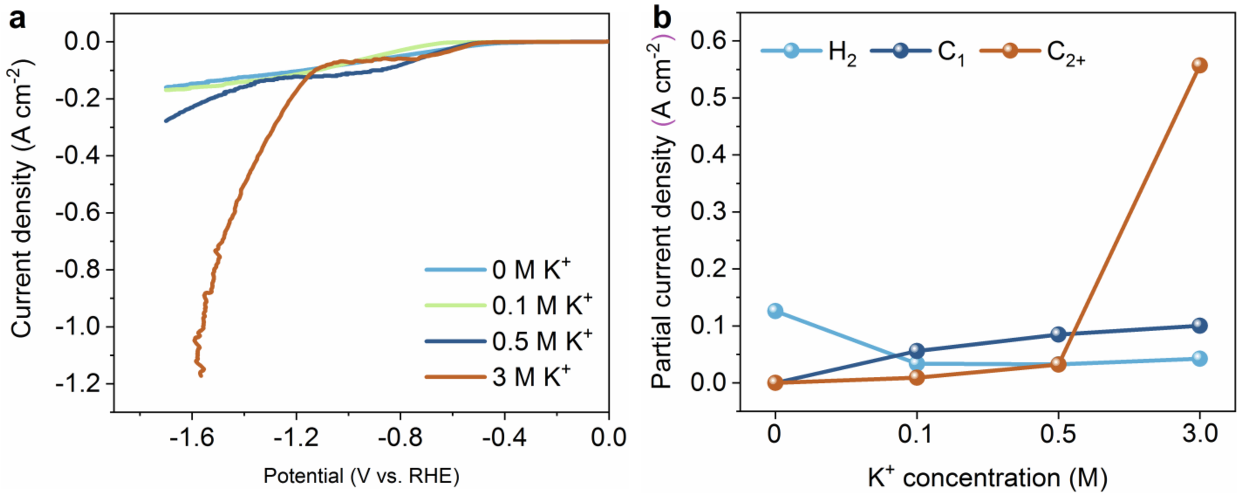

Supplementary Fig. 15 (a) CO_2RR LSV curves and partial current density for ER-CuNS in 0.05 M H_2SO_4 with different amount of K^+ additives.

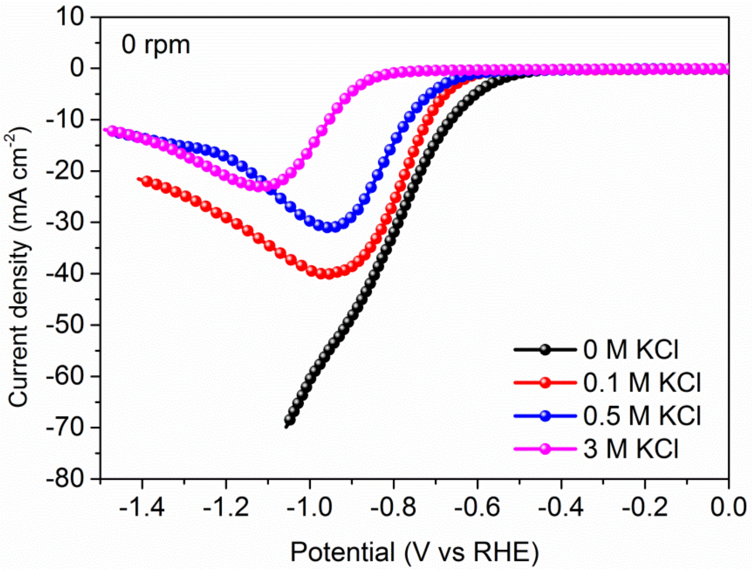

Supplementary Fig. 16 HER LSV curves at 0 rpm for ER-CuNS in 0.05 M H_2SO_4 with different amount of KCl.

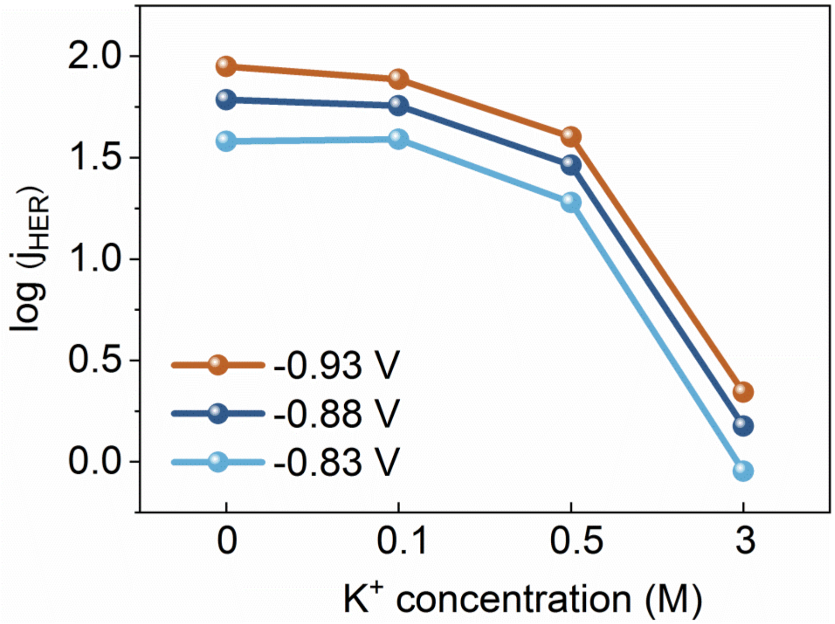

Supplementary Fig. 17 HER current density at 0 rpm of ER-CuNS in 0.05 M H_2SO_4 with different K^+ concentrations.

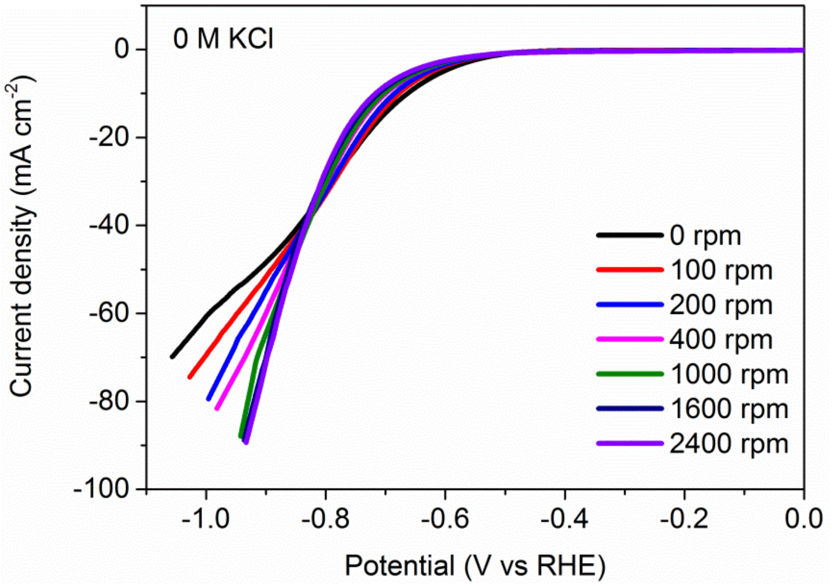

Supplementary Fig. 18 HER LSV curves at varying rotating speed for ER-CuNS in 0.05 M H_2SO_4.

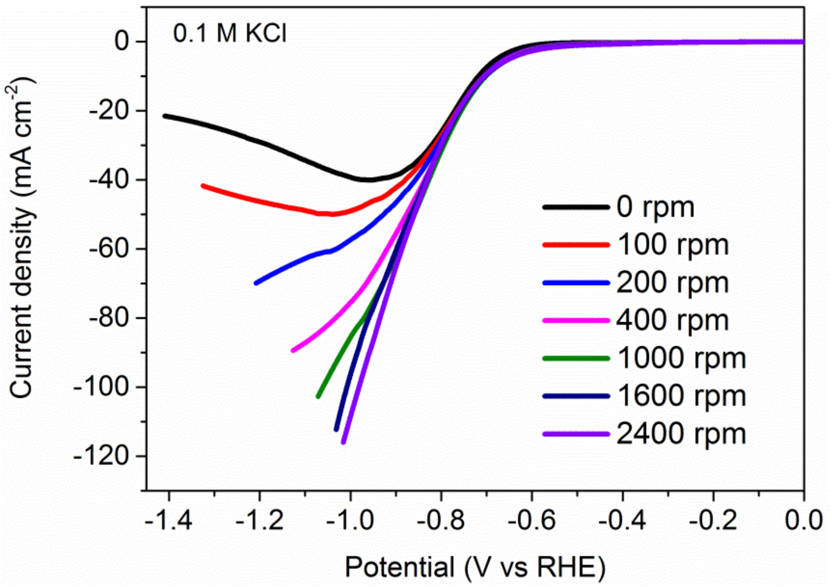

Supplementary Fig. 19 HER LSV curves at varying rotating speed for ER-CuNS in 0.05 M H_2SO_4 with 0.1 M KCl.

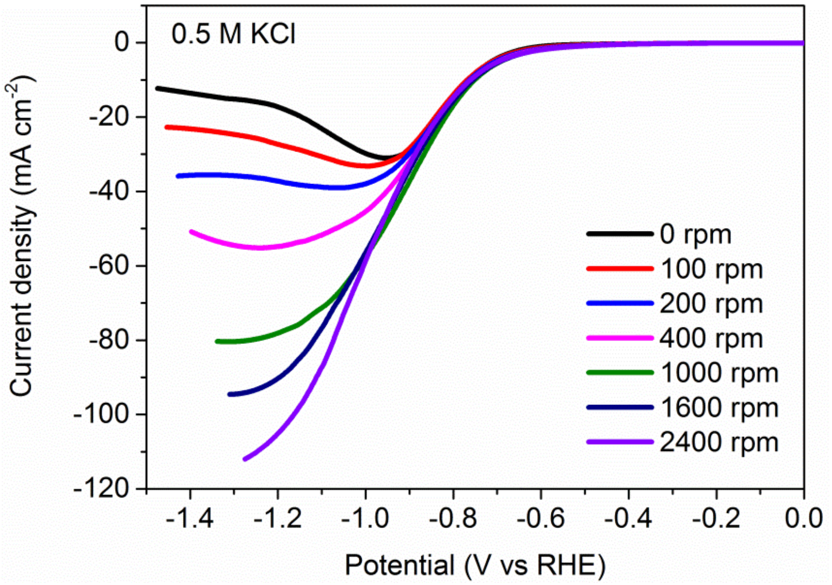

Supplementary Fig. 20 HER LSV curves at varying rotating speed for ER-CuNS in 0.05 M H_2SO_4 with 0.5 M KCl.

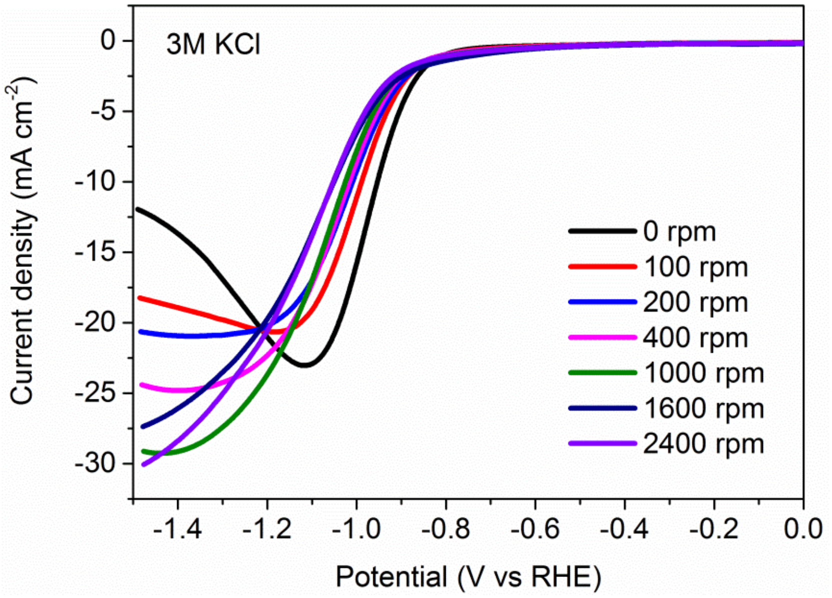

Supplementary Fig. 21 HER LSV curves at varying rotating speed for ER-CuNS in 0.05 M H_2SO_4 with 3 M KCl.

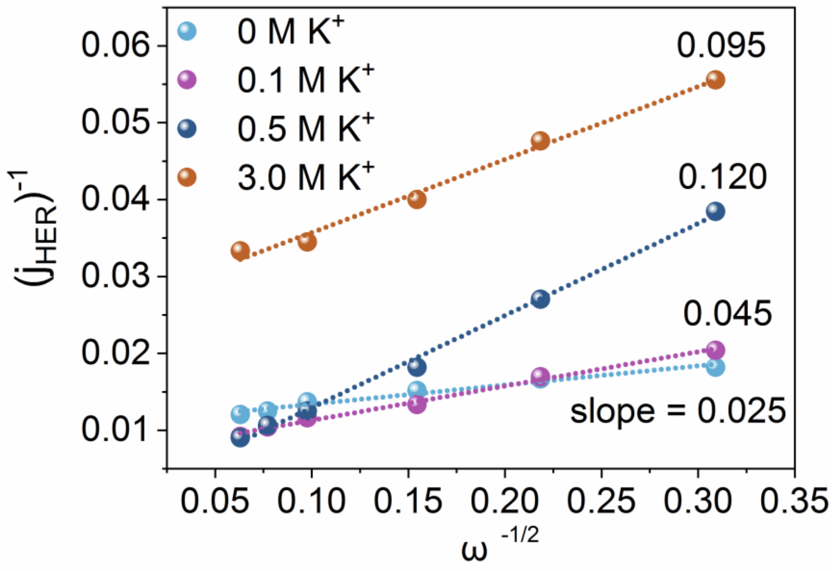

Supplementary Fig. 22 The fitting of $(j_{HER})^{-1}$ *vs* $\omega^{-1/2}$ obtained from HER data in acid with different K^+ concentrations

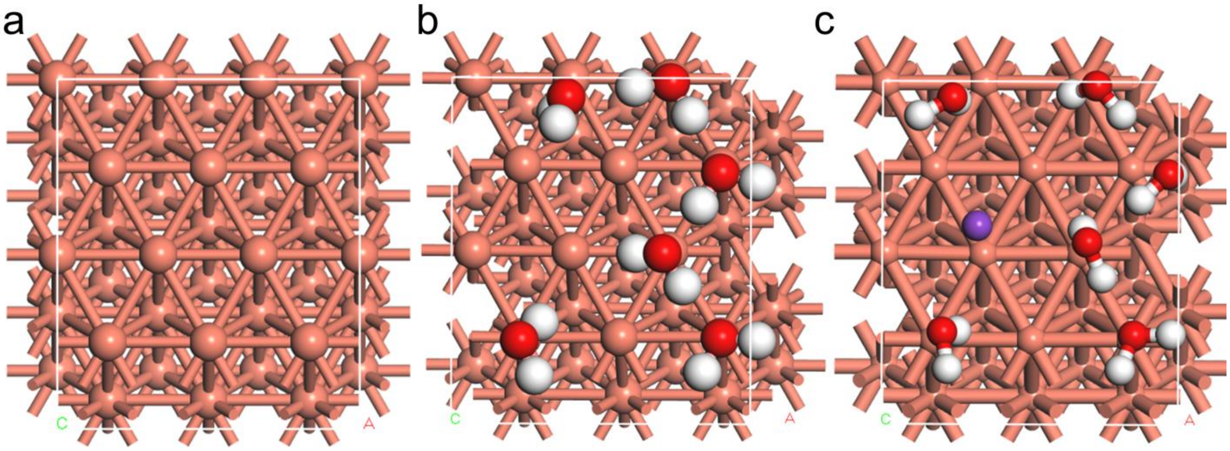

Supplementary Fig. 23 The top-view models for DFT calculations. (a) Cu (111) slab, (b) Cu (111) slab with H_2O, and (c) Cu (111) slab with H_2O and K^+ species on surface. The bronze, red, white and purple balls represent Cu, O, H and K atoms, respectively.

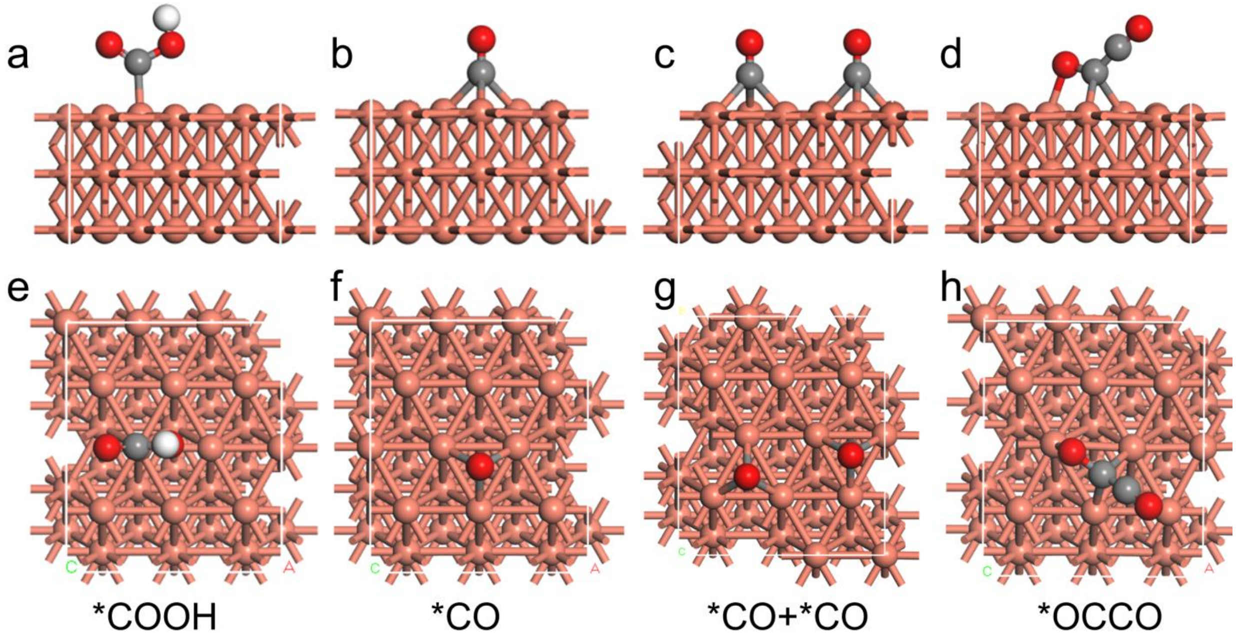

Supplementary Fig. 24 The configurations of CO_2RR pathways on Cu (111) slab. (a, e) *COOH configuration. (b, f) *CO configuration. (c, g) two *CO on slab. (d, h) *COCO configuration after *CO dimerization. (a-d) side view, (e-h) top view. The bronze, grey, red and white balls represent Cu, C, O and H atoms, respectively.

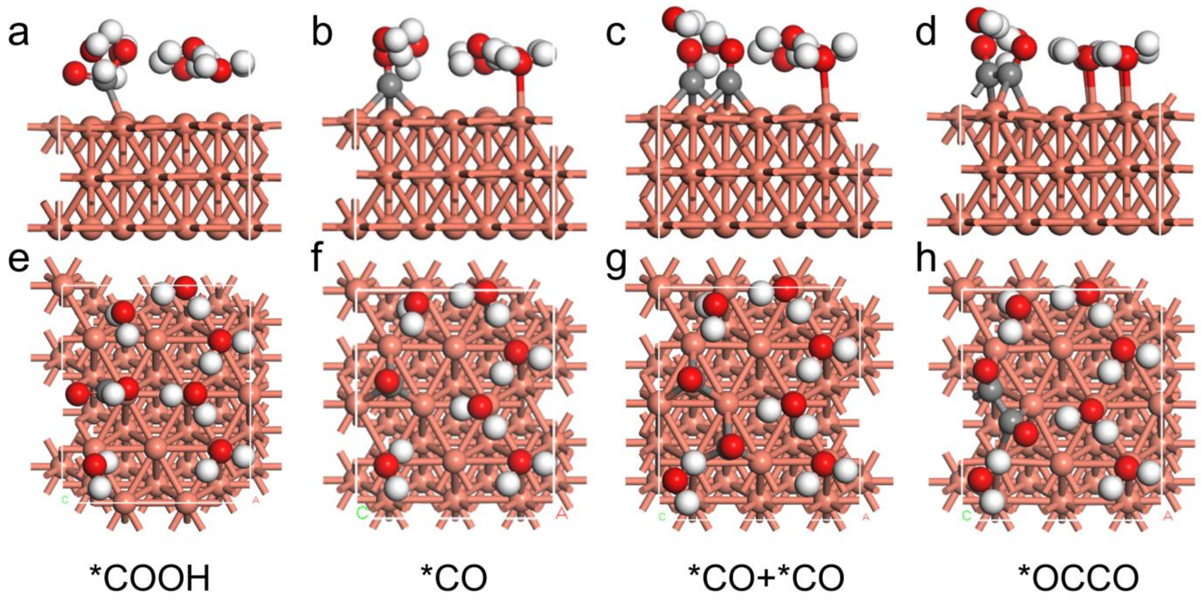

Supplementary Fig. 25 The configurations of CO_2RR pathways on Cu (111) slab with H_2O. (a, e) *COOH configuration. (b, f) *CO configuration. (c, g) two *CO on slab. (d, h) *COCO configuration after *CO dimerization. (a-d) side view, (e-h) top view. The bronze, grey, red and white balls represent Cu, C, O and H atoms, respectively.

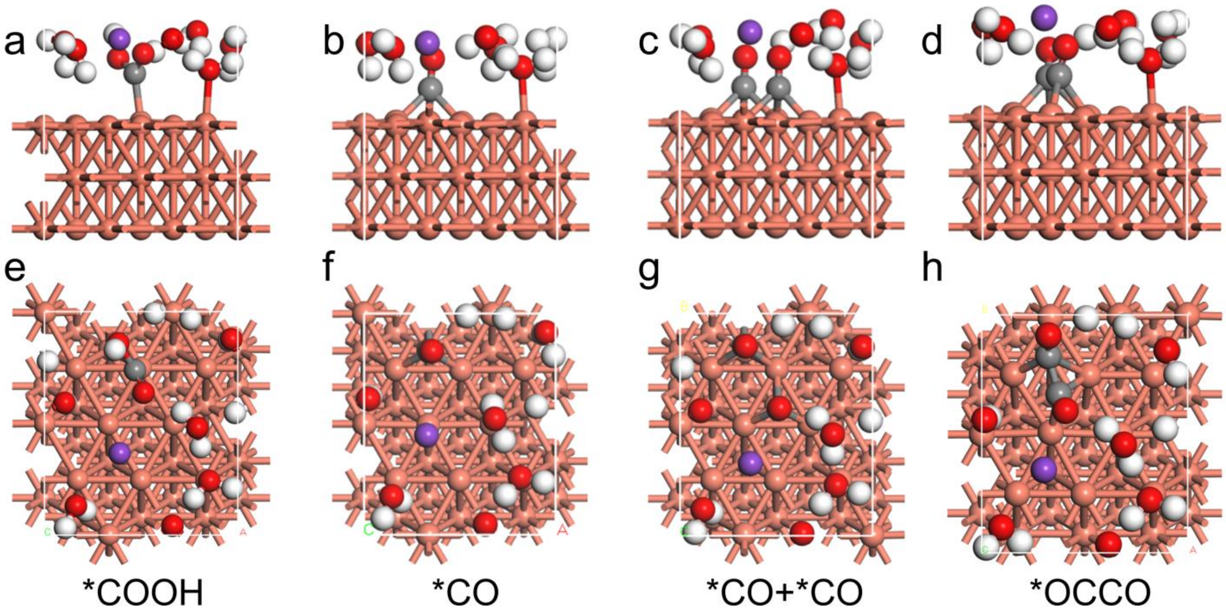

Supplementary Fig. 26 The configurations of CO_2RR pathways on Cu (111) slab with H_2O and K^+ species. (a, e) *COOH configuration. (b, f) *CO configuration. (c, g) two *CO on slab. (d, h) *COCO configuration after *CO dimerization. (a-d) side view, (e-h) top view. The bronze, grey, red, white and purple balls represent Cu, C, O, H and K atoms, respectively.

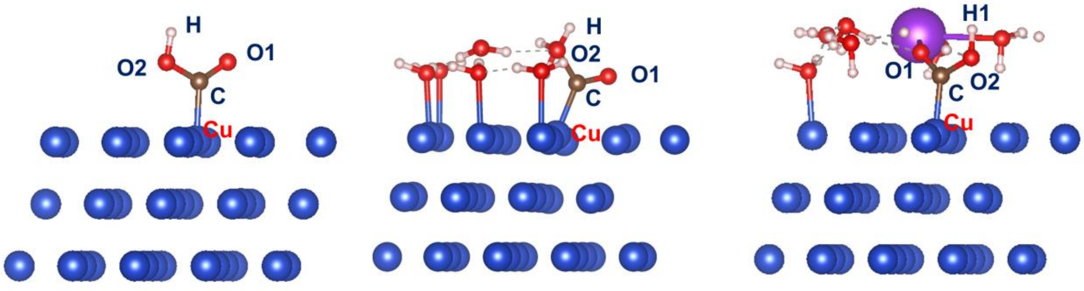

Slab with *COOH	Cu	Cu-H_2O	Cu-H_2O-K^+
**Cu**	10.954357	10.894110	10.890516
**C**	2.726563	2.864738	2.830384
**O1**	7.101796	7.152919	7.214230
**O2**	7.088049	7.133080	7.135806
**H**	0.419593	0.390817	0.405492

Supplementary Fig. 27 The Bader charge analysis for *COOH on different slabs. The blue, brown, red, white and purple balls represent Cu, C, O, H and K atoms, respectively.

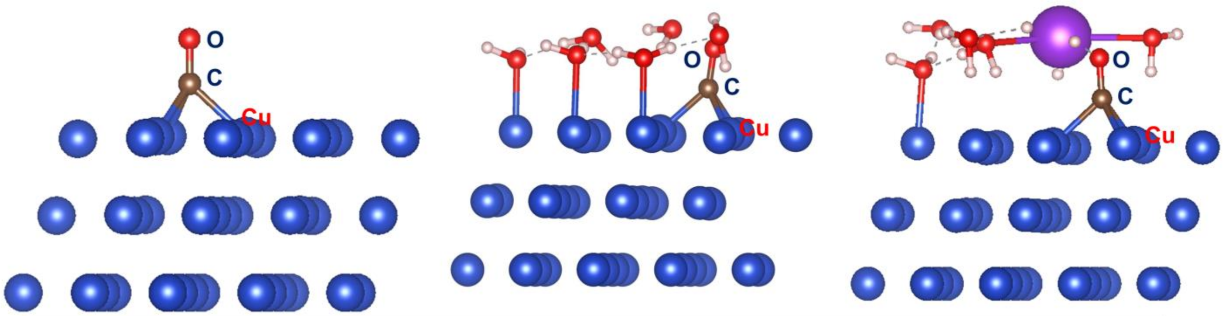

Slab with *CO	Cu	Cu-H_2O	Cu-H_2O-K^+
Cu	10.954357	10.894110	10.915189
C	2.726563	2.864738	3.524774
O	7.088049	7.133080	7.039106

Supplementary Fig. 28 The Bader charge analysis for *CO on different slabs. The blue, brown, red, white and purple balls represent Cu, C, O, H and K atoms, respectively.

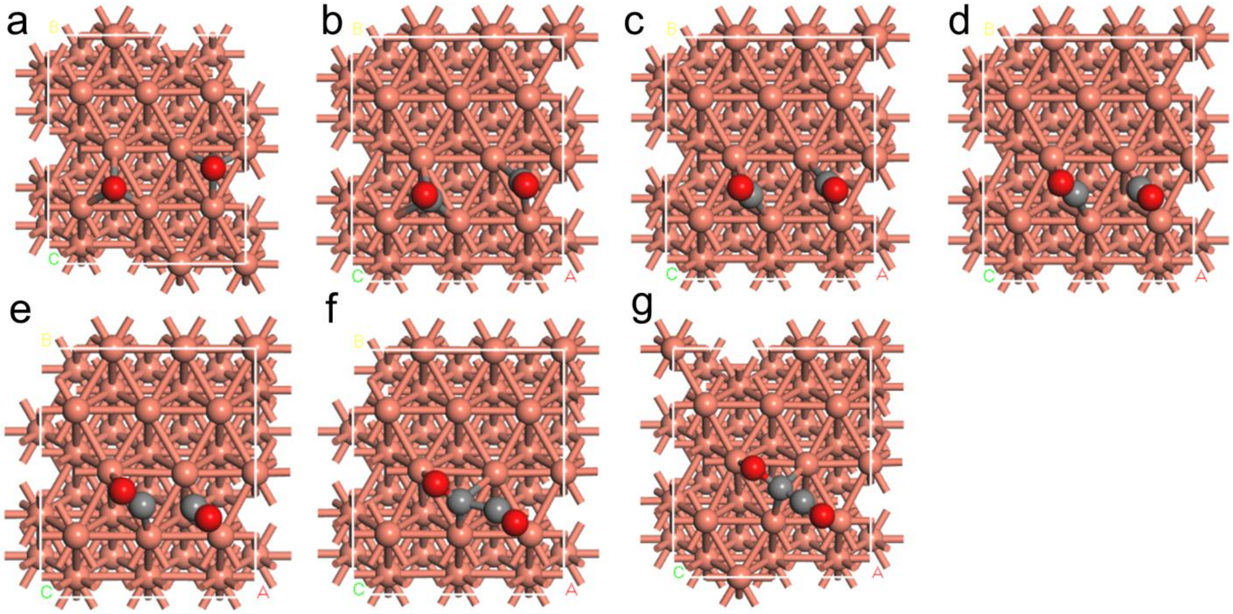

Supplementary Fig. 29 The configurations along CO dimerization pathway on Cu (111) slab from top view. The bronze, grey and red balls represent Cu, C and O atoms, respectively.

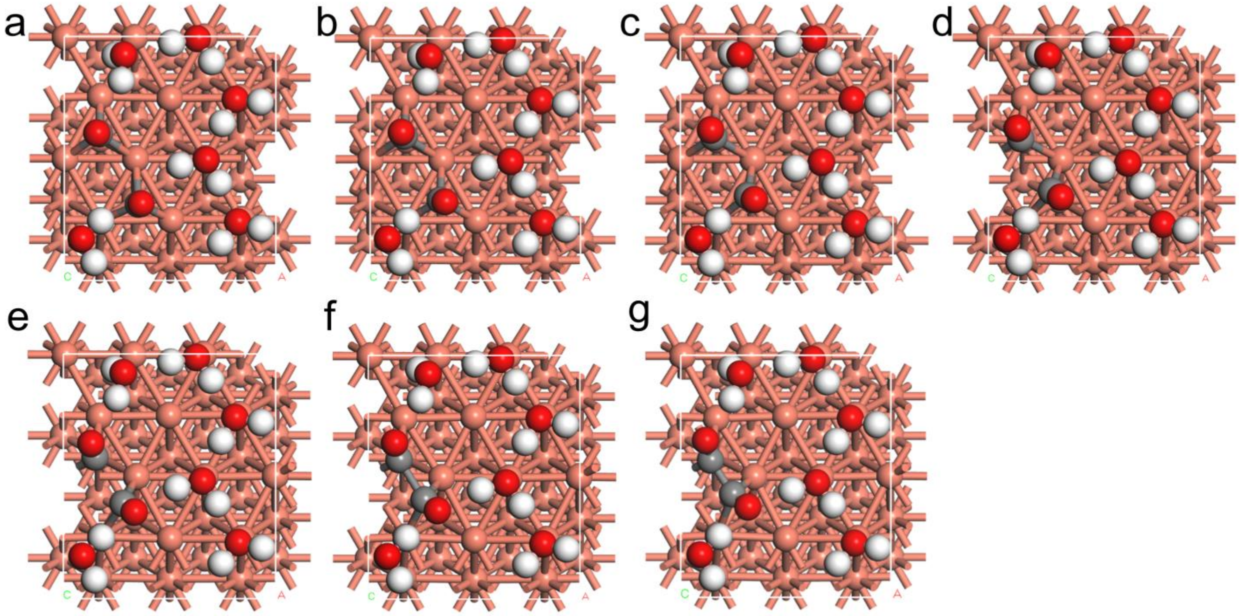

Supplementary Fig. 30 The configurations along CO dimerization pathway on Cu (111) slab with H_2O from top view. The bronze, grey, red and white balls represent Cu, C, O and H atoms, respectively.

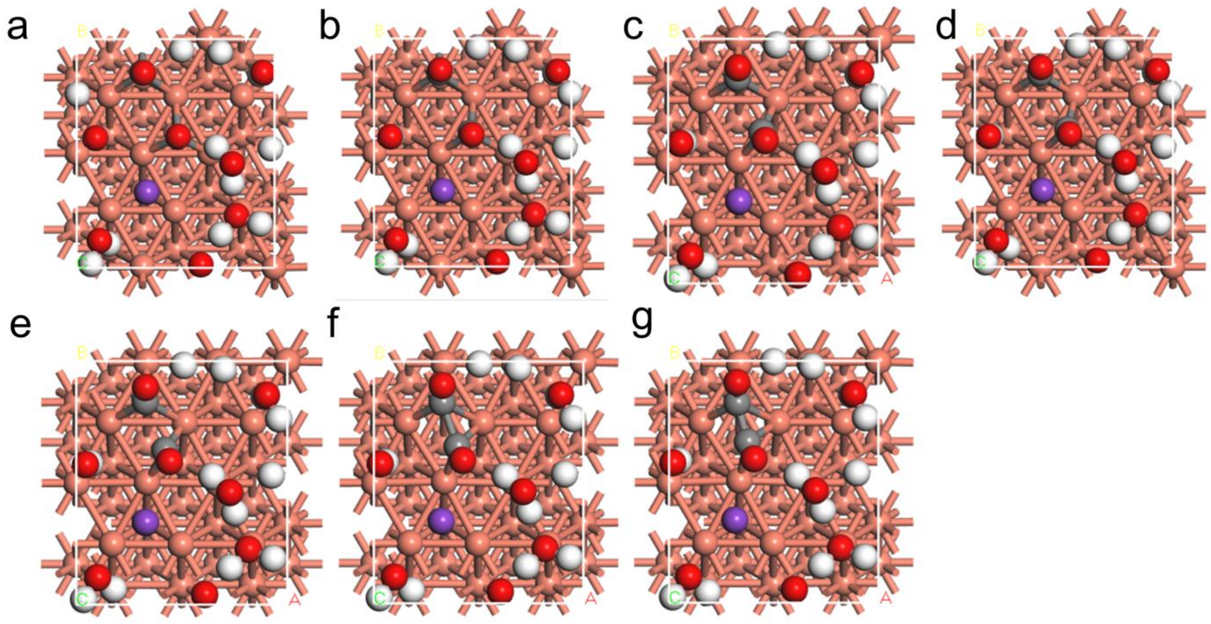

Supplementary Fig. 31 The configurations along CO dimerization pathway on Cu (111) slab with H_2O and K^+ species from top view. The bronze, grey, red, white and purple balls represent Cu, C, O, H and K atoms, respectively.

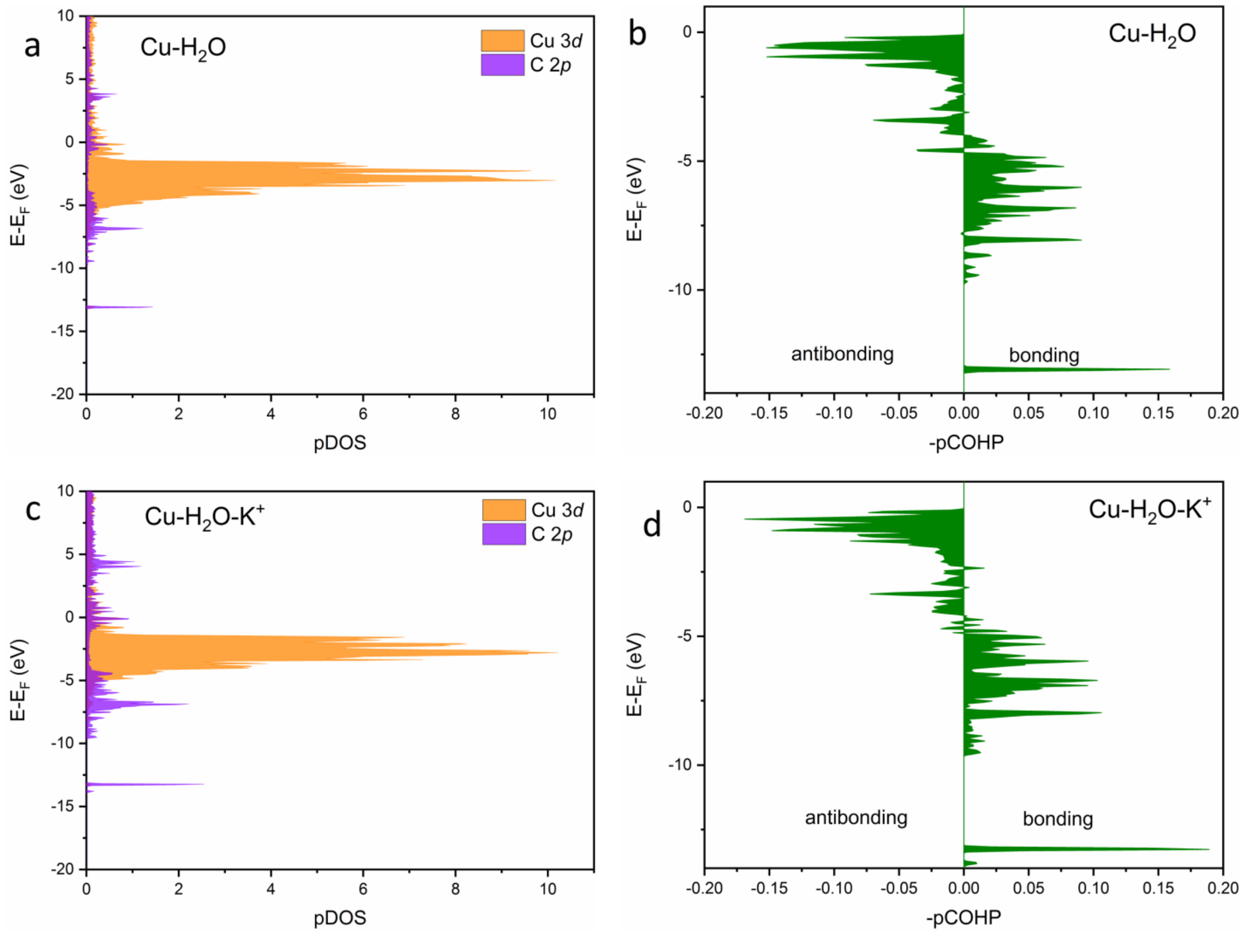

Supplementary Fig. 32 Projected density of states (pDOS) and projected crystal orbital Hamilton populations (-pCOHP) for the Cu-C bond of *OCCO bonded to the Cu-H_2O surface without/with K^+ cation.

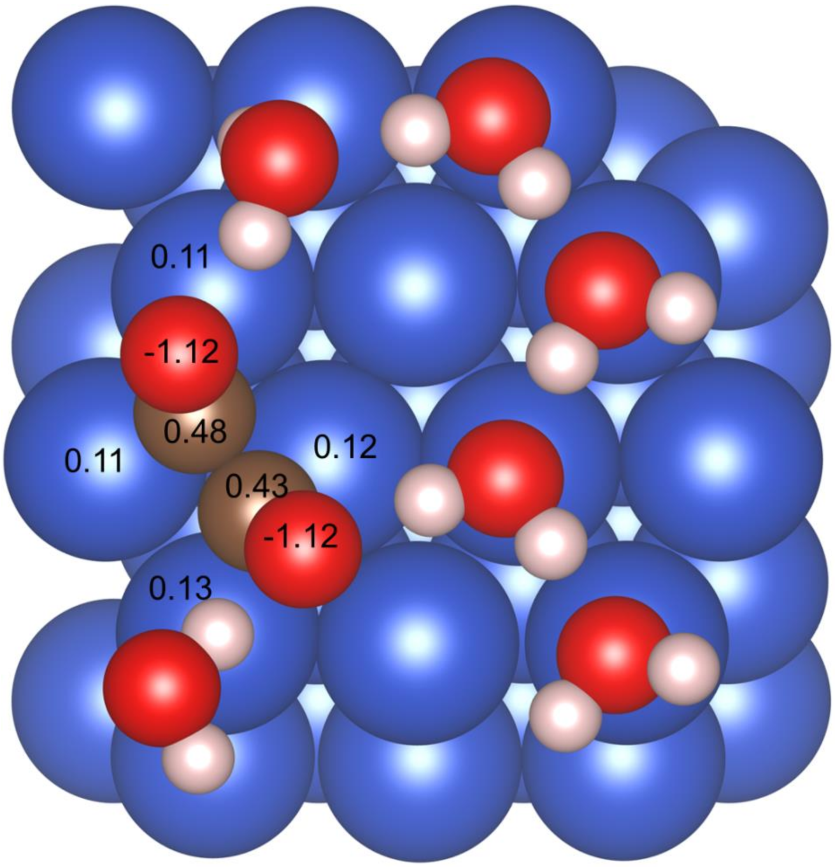

Supplementary Fig. 33 The charge density analysis of *OCCO on Cu-H_2O slab derived from the calculated Bader charge. The blue, brown, red and white balls represent Cu, C, O and H atoms, respectively.

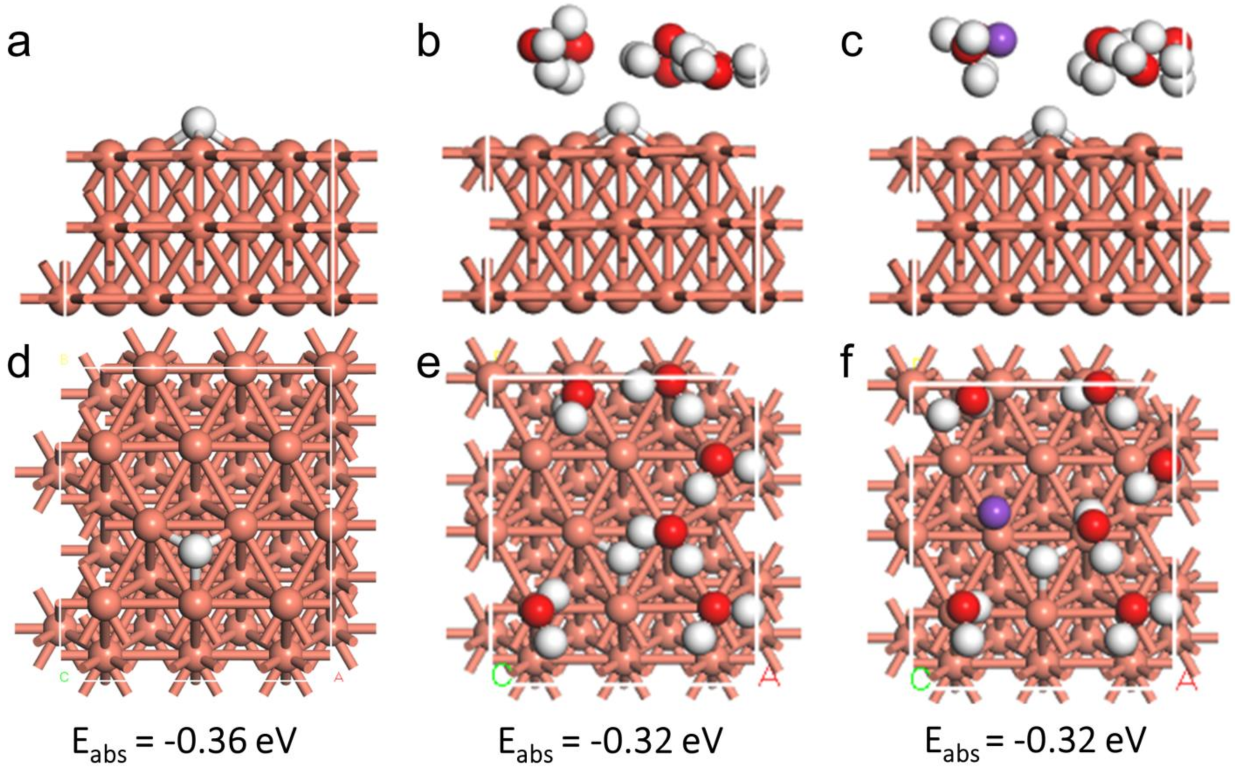

Supplementary Fig. 34 The configurations of hydrogen adsorption on different slabs. (a, d) Cu (111) slab, (b, e) Cu (111) slab with H_2O, and (c, f) Cu (111) slab with H_2O and K^+ species on surface. (a-c) side view, (d-f) top view. The hydrogen adsorption energies are given at the bottom. The bronze, red, purple and white balls represent Cu, O, K and H atoms, respectively.

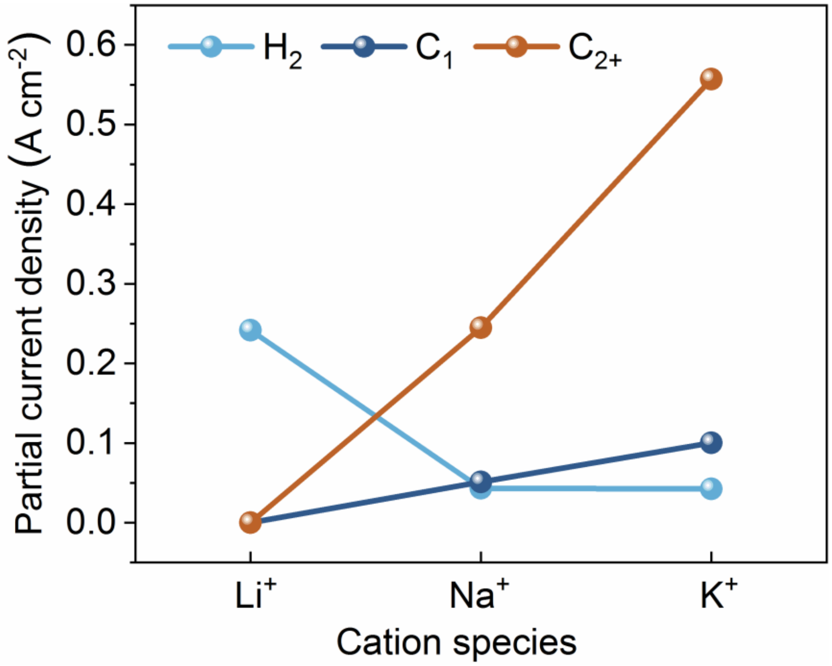

Supplementary Fig. 35 CO_2RR partial current density for ER-CuNS in 0.05 M H_2SO_4 with 3 M alkaline metal cations additives at -1.45 V_{RHE}.

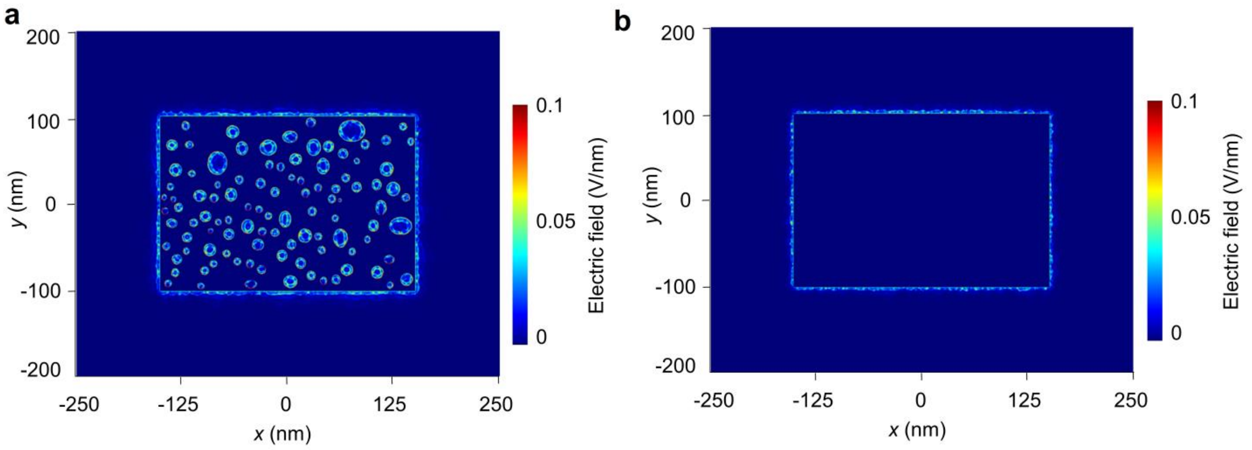

Supplementary Fig. 36 Finite difference time domain simulations of electric fled distribution on ER-CuNS and F-CuNS.

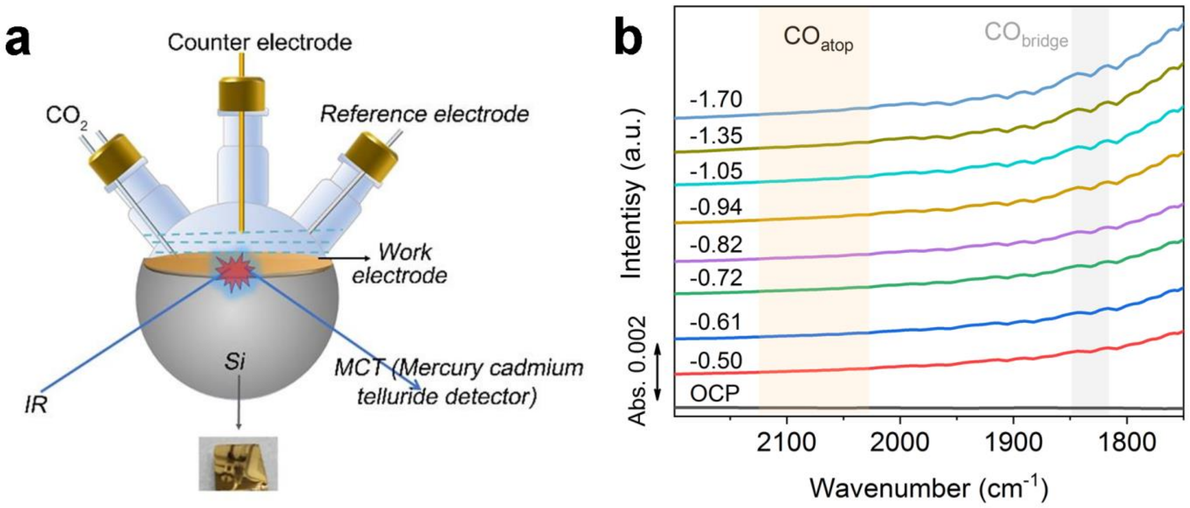

Supplementary Fig. 37 (a) Schematic representation of attenuated total reflection surface-enhanced infrared absorption spectroscopy (ATR-SEIRAS). (b) In situ ATR- SEIRAS spectra of acidic CO_2RR on ER-CuNS collected under different applied potentials (V vs RHE) in 0.05 M H_2SO_4 electrolyte.

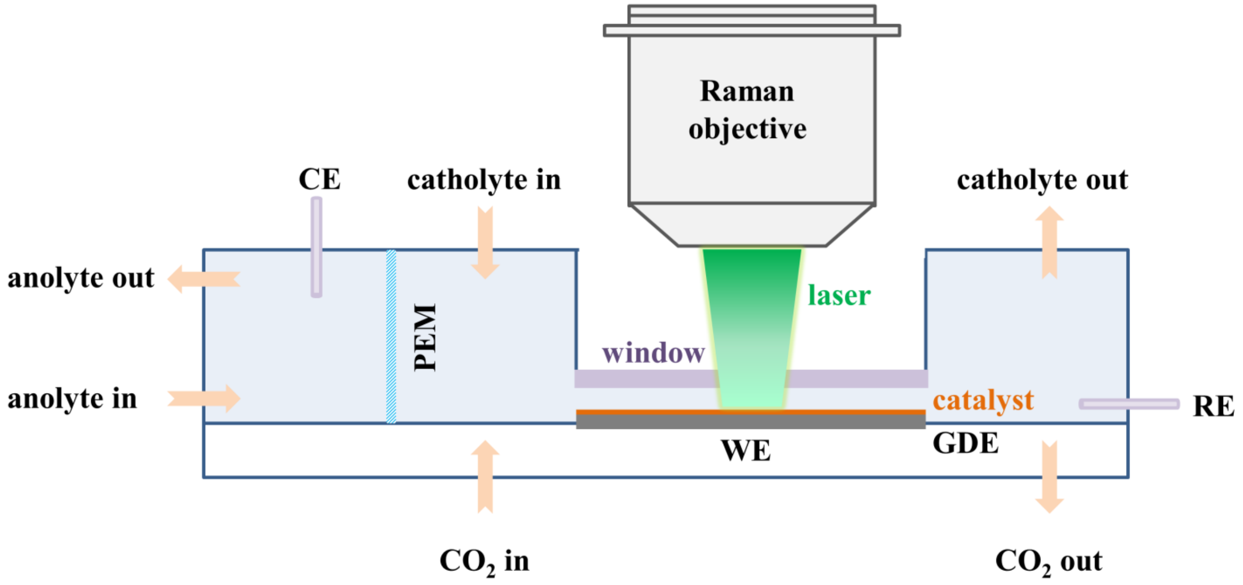

Supplementary Fig. 38 Schematic representation of in-situ Raman measurements. The catholyte was 0.05 M H_2SO_4 with 3 M KCl additives, and anolyte was 0.05 M H_2SO_4 solution. ER-CuNS and F-CuNS catalysts were loaded on the carbon paper GDE, and CO_2 was supplied from the back side of GDE. Because of the different design in electrolyzer, carbon-based GDE instead of PTFE-based GDE was used here for current collecting.

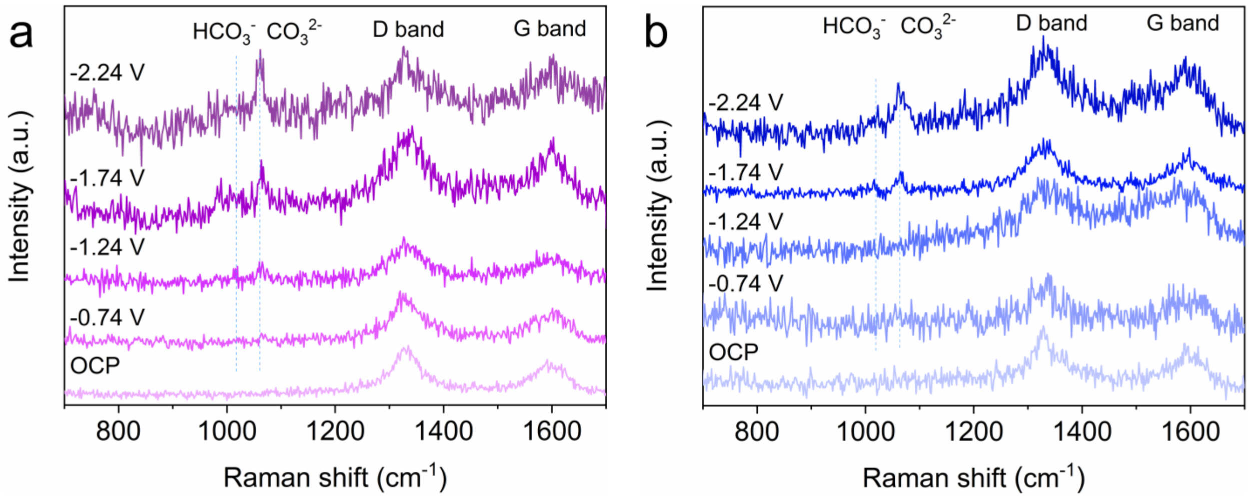

Supplementary Fig. 39 In-situ Raman spectra of acidic CO_2RR on (a) ER-CuNS and (b) F-CuNS at different potentials (vs RHE, no iR correction). The D band and G band of carbon electrode are also presented for reference. The HCO_3^- peak locates at around 1016 cm^{-1}, while CO_3^{2-} peak at around 1065 cm^{-1}.[1,2]

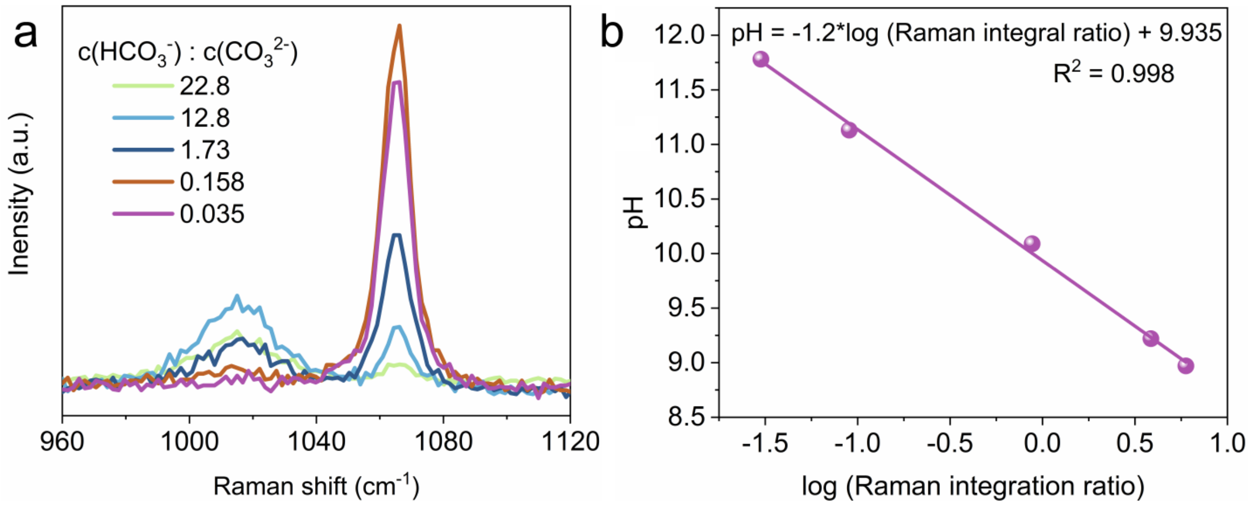

Supplementary Fig. 40 (a) The Raman spectra for mixed HCO_3^-/CO_3^{2-} standard solutions with different ion concentration ratios ($c(HCO_3^-)/c(CO_3^{2-})$). (b) Linear fitting between pH and log (Raman integration ratio) based on Raman spectra of standard solutions (Supplementary Table 3).

Supplementary Note S1. Method to calculate local pH based on Raman signal of HCO_3^- and CO_3^{2-} species at electrode-electrolyte interface under CO_2RR working conditions.

Supplementary Fig. 39 gives the Raman spectra of reaction interface for ER-CuNS and F-CuNS catalyst under working conditions. At open-circuit potential (OCP) state where no electrochemical reactions happen, the local pH at electrode-electrolyte interface is basically same as bulk electrolyte (pH ≤ 1). Therefore, no signal of HCO_3^- or CO_3^{2-} can be detected even in the presence of CO_2 reactant due to the weak ionization of H_2CO_3 at pH ≤ 1. With potential becoming more negative and electrochemical reactions starting, the depletion of H^+ or generation of OH^- species by HER or CO_2RR, gradually creates the local alkaline microenvironment. The reaction equilibrium of H_2CO_3 thus shifts (Eq. 1 and 2, *K* stands for equilibrium constant), resulting in more and detectable HCO_3^-/CO_3^{2-} species at the interface. As shown in Supplementary Fig. 39, the signals of HCO_3^-/CO_3^{2-} appear at -1.24 V_{RHE}, hinting the generation of local alkaline environment for ER-CuNS, while more negative potential (-1.74 V_{RHE}) is required to create the interfacial alkalinity for F-CuNS.

$$H_2CO_3 + OH^- \overset{K_1}{\rightleftharpoons} HCO_3^- + H_2O \quad \text{(Eq. 1)}$$

$$HCO_3^- + OH^- \overset{K_2}{\rightleftharpoons} CO_3^{2-} + H_2O \quad \text{(Eq. 2)}$$

$$H_2O \overset{K_3}{\rightleftharpoons} H^+ + OH^- \quad \text{(Eq. 3)}$$

We then turn to the quantification of local pH by referring the work of Zhang et al. but with some modifications considering the difference between two systems.[2] Fundamentally, based on reaction equilibrium in Eq. 2 and 3, the pH can be given as Eq. 4 as follows:

$$pH = -\log(a_{(H+)}) = -\log(\frac{K_3}{a_{(OH-)}}) = -\log(\frac{K_3K_2a_{(HCO_3^-)}}{a_{(CO_3^{2-})}}) = -\log(K_3K_2) - \log(\frac{\gamma_{(HCO_3^-)}}{\gamma_{(CO_3^{2-})}}) - \log(\frac{c_{(HCO_3^-)}}{c_{(CO_3^{2-})}}) \quad \text{(Eq. 4)}$$

where $a(H^+)$, $a(OH^-)$, $a(HCO_3^-)$ and $a(CO_3^{2-})$ stand for activity of H^+, OH^-, HCO_3^- and CO_3^{2-} species, $\gamma(HCO_3^-)$ and $\gamma(CO_3^{2-})$ respectively mean the activity coefficient of HCO_3^- and CO_3^2, $c(HCO_3^-)$ and $c(CO_3^{2-})$ are the corresponding molar concentration. To simplify the calculations, the activity coefficient is approximately regarded as the constant and separated from the $c(HCO_3^-)/c(CO_3^{2-})$ variable.[1] As a result, it is demonstrated by Eq. 4 that basically, pH has a linear correlation with molar concentration ratio between HCO_3^- and CO_3^{2-} ($c(HCO_3^-)/c(CO_3^{2-})$).[3,4] Experimentally, to get the specific correlation between pH and $c(HCO_3^-)/c(CO_3^{2-})$ value, we prepared the mixed HCO_3^-/CO_3^{2-} standard solutions with different ion concentration ratios and collected their Raman spectra (Supplementary Fig. 40a).

Supplementary Table 3 summarizes the detailed $c(HCO_3^-)/c(CO_3^{2-})$, Raman integration ratio between HCO_3^- and CO_3^{2-}, and pH values of standard solutions determined by pH meter. By fitting the pH value against log (Raman integration ratio), we obtained the linear relationship ass shown in Supplementary Fig. 40b, consistent with the analysis from Eq. 4. Note that log (Raman integration ratio) instead of log ($c(HCO_3^-)/c(CO_3^{2-})$), was directly used for fitting. This is reasonable because log (Raman integration ratio) was also linearly correlated with log $c(HCO_3^-)/c(CO_3^{2-})$ in Supplementary Table 3. By using the linear equation in Supplementary Fig. 40b, we then estimated the local pH values for acidic CO_2RR on ER-CuNS and F-CuNS based on Raman peak integration ratio in Supplementary Fig. 39. As shown in Supplementary Table 4, the ER-CuNS electrode behaves higher local pH, namely higher local OH^- concentration, compared to F-CuNS at the same potentials.

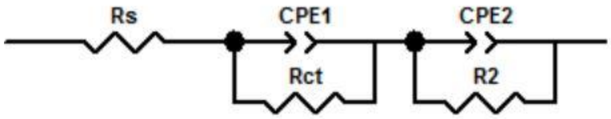

Supplementary Scheme S1. The equivalent circuit for fitting EIS plots in Supplementary Fig. 11-12, where R_S means solution resistance and R_{ct} is interfacial charge transfer resistance. The constant phase element (CPE) was necessarily introduced to account for the nonideality of the interface between the electrode and electrolyte in the practical impedance spectrum. More specifically, the CPE1 and CPE2 are the bulk-electrolyte and the double-layer capacitances. R_{ct1} and R_{ct2} represent the charge-transfer resistance at the electrocatalyst film and electrocatalyst film/electrolyte interface, respectively, which indicates the difficult degree of kinetic reaction in the electrocatalytic process.[5]

Supplementary Table 1. The fitted parameters of the EIS plots in Supplementary Fig. 11-12 for ER-CuNS or F-CuNS in 0.05 M H_2SO_4 electrolyte with different molar concentration of KCl.

Catalyst	c [KCl]	R_S (Ω)	CPE1-T (F s^{n-1})	CPE1-P	R_{ct1} (Ω)	CPE2-T (F s^{n-1})	CPE2-P	R_{ct2}(Ω)
F-CuNS	3 M	9.7	0.002686	0.44820	5.3	0.016117	0.57797	810.2
ER-CuNS	3 M	5.8	0.050953	0.84097	4.0	0.063349	0.67254	53.6
ER-CuNS	0.5 M	17.8	0.006652	0.81529	40.3	0.021058	0.75797	202.0
ER-CuNS	0.1 M	41.1	0.000909	0.97835	172.4	0.003816	0.70321	279.7
ER-CuNS	0 M	70.1	0.000339	0.78436	197.5	0.097063	0.32262	325.6

Supplementary Table 2. A brief comparison between our catalyst and ever-reported catalysts that aim at manufacturing C_{2+} products in acidic CO_2RR system.

Ref	pH	C_{2+} selectivity	C_{2+} current density	Durability
Our work	0.51	84%	557 mA cm^{-2}	30 h
Huang et al.[6]	0.67	48%	576 mA cm^{-2}	12.5 h
Xie et al.[7]	2.0	89%	500 mA cm^{-2}	4.5 h
Gu et al.[8]	~0.3	~37%	~200 mA cm^{-2}	4 h

Supplementary Table 3. The Raman integration ratio (and its logarithm) and pH (determined by pH meter) for mixed HCO_3^-/CO_3^{2-} standard solutions with different molar concentration ratios. The Raman data is derived from Supplementary Fig. 40.

$c(HCO_3^-)/c(CO_3^{2-})$	log $(c(HCO_3^-)/c(CO_3^{2-}))$	Raman integration ratio	log (Raman integration ratio)	pH
22.8	1.3579	5.97	0.77597	8.97
12.8	1.1079	3.86	0.58659	9.22
1.73	0.2379	0.88	-0.05552	10.09
0.158	-0.8021	0.09	-1.04576	11.13
0.035	-1.4521	0.03	-1.52288	11.78

Supplementary Table 4. Calculated local pH on ER-CuNS and F-CuNS surface based on in-situ Raman spectra of acidic CO_2RR system in Supplementary Fig. 39.

Sample	Potential (V vs Ag/AgCl)	Raman integration ratio	Calculated local pH
**ER-CuNS**	OCP	/	/
	-1.0	/	/
	-1.5	0.71	10.11
	-2.0	0.21	10.74
	-2.5	0.07	11.36
**F-CuNS**	OCP	/	/
	-1.0	/	/
	-1.5	/	/
	-2.0	0.77	10.07
	-2.5	0.35	10.49

Supplementary References

1. Lu, X. *et al.* In Situ Observation of the pH Gradient near the Gas Diffusion Electrode of CO_2 Reduction in Alkaline Electrolyte. *J. Am. Chem. Soc.* **142**, 15438-15444 (2020).
2. Zhang, Z. *et al.* pH Matters When Reducing CO_2 in an Electrochemical Flow Cell. *ACS Energy Lett.* **5**, 3101-3107 (2020).
3. Jiang, S., Klingan, K., Pasquini, C. & Dau, H. New aspects of operando Raman spectroscopy

applied to electrochemical CO_2 reduction on Cu foams. *J. Chem. Phys.* **150**, 041718 (2018).

4. Hills, A. G. pH and the Henderson-Hasselbalch equation. *Am. J. Med.* **55**, 131-133 (1973).

5. Jiang, X. *et al.* A highly selective tin-copper bimetallic electrocatalyst for the electrochemical reduction of aqueous CO_2 to formate. *Appl. Catal. B: Environ.* **259**, 118040 (2019).

6. Huang Jianan, E. *et al.* CO_2 electrolysis to multicarbon products in strong acid. *Science* **372**, 1074-1078 (2021).

7. Xie, Y. *et al.* High carbon utilization in CO_2 reduction to multi-carbon products in acidic media. *Nat. Catal.* **5**, 564-570 (2022).

8. Gu, J. *et al.* Modulating electric field distribution by alkali cations for CO_2 electroreduction in strongly acidic medium. *Nat. Catal.* **5**, 268-276 (2022).
